# Region-specific quantitation of glycosphingolipids in the elderly human brain with Nanoflow MEA Chip Q/ToF mass spectrometry

**DOI:** 10.1093/glycob/cwaf022

**Published:** 2025-04-10

**Authors:** Ryan L Schindler, Lee-way Jin, Angela M Zivkovic, Yiyun Liu, Carlito B Lebrilla

**Affiliations:** Department of Chemistry, University of California, Davis, CA 95616, United States; Department of Pathology and Laboratory Medicine, University of California Davis Medical Center, Sacramento, CA 95616, United States; Department of Nutrition, University of California, Davis, CA 95616, United States; Department of Chemistry, University of California, Davis, CA 95616, United States; Department of Chemistry, University of California, Davis, CA 95616, United States

**Keywords:** brain map, glycosphingolipids, MEA Chip, Nanoflow HPLC-Q, ToF

## Abstract

Glycosphingolipids are a unique class of bioactive lipids responsible for lateral membrane organization and signaling found in high abundance in the central nervous system. Using nanoflow MEA Chip Q/ToF mass spectrometry, we profiled the intact glycosphingolipids of the elderly human brain in a region-specific manner. By chromatographic separation of glycan and ceramide isomers, we determined gangliosides to be the highest source of heterogeneity between regions with the expression of a- and b-series glycan structures. Investigation of these trends showed that specific glycan structures were, in part, determined by the structure of their lipid backbone. This study provides insight into the dynamic process of membrane remodeling in the brain during aging.

## Introduction

Significant scientific progress has been made to profile the biochemical makeup of the brain to elucidate structure-to-function neurophysiology and pathology. Curated databases are available for the transcriptome (Keil et al. 2018) and proteome (Lam et al. 2022) of the human brain on a regional and cellular level. However, there is not a comparable resource for the lipidome. Using a single-cell lipidomic workflow, Bhaduri et al. observed comparable profiles in the most abundant lipids between region-matched human brains but heterogeneity when making regional and age-matched comparisons (Bhaduri et al. 2021). This work, along with others, emphasized the importance of lipids in the central nervous system (CNS), which may be vital to understanding neurological mechanisms and developing therapeutics for various forms of neurodegeneration.

Sphingolipids (SL) are a subset of bioactive lipids found in eukaryotic cell membranes and are unique in their amphipathic structure and potential to be glycosylated (Merrill 2011). The human nervous system contains a significantly higher abundance of glycosphingolipids (GSLs) than the other systems, indicating their physiological importance. A recent study by Blumenreich et al. showed that increased ganglioside expression (Sialic acid containing GSLs) was correlated to subjects with a genetic risk factor for Parkinson’s disease in three of the four regions studied (Blumenreich et al. 2022). Gangliosides have been indicated to play a role in the pathology of Huntington’s disease, amyotrophic lateral sclerosis, Alzheimer’s disease, stroke, multiple sclerosis, and epilepsy (Sipione et al. 2020).

SLs are characterized by their two-tailed lipid backbone—a ceramide consisting of a sphingoid base and an N-linked acyl group – that is enzymatically bound by one of a family of ceramide synthases (Merrill 2011). Ceramides tend to have longer, more saturated lipid tails than other major membrane lipids. Along with the ceramide’s structural contribution to membrane biophysical properties, they play a crucial role in cellular homeostasis by regulating apoptosis and autophagy (Young et al. 2013). The bioactive head group of SLs extends from the outer membrane into the extracellular space, mediating interactions such as signaling, adhesion, and differentiation (Fernandis and Wenk 2007; D’Angelo et al. 2013). Unlike other membrane lipids, SLs are unique in their ability to contain oligosaccharide (glycan) headgroups. The untemplated biosynthesis of these glycan headgroups is a dynamic process involving families of glycosyltransferases and glycosidases, which fine-tune the glycocalyx and subsequent intra- and inter-cellular interactions (Schnaar et al. 2022). Subtle structural changes such as the position of a sialic acid (Neu5Ac) or the stereochemistry of a glycosidic bond (α or β) can have significant effects on carbohydrate-receptor binding and overall cellular phenotype (van Kooyk and Van Kooyk and Rabinovich 2008).

Although constituting a small fraction of the overall lipid profile, SL’s contribution to cellular biophysical properties is thought to be spatially amplified due to clustering with proteins and cholesterol into microdomains commonly referred to as lipid rafts (Todeschini and Hakomori 2008). This clustering occurs due to a high degree of hydrogen bonding from the polar moieties and ordered stacking of the lipid backbones. These SL-rich microdomains are categorized by a highly ordered “solid-like” membrane phase with a slow translational diffusion coefficient (Mencarelli and Martinez-Martinez 2013). These rafts translocate in the bulk liquid-disordered bilayer, self-assembling into functional domains that carry out essential cellular functions such as synaptic transmission in neurons.

General profiling of GSLs in the brain has revealed that white matter is enriched in Gal-Cer and SGal-Cer, where they pair to form carbohydrate-carbohydrate bonds, providing intra-membrane stability to the multi-layered myelin sheath (Boggs et al. 2008). Grey matter, consisting primarily of neuronal cell bodies and axons, is highly expressed with gangliosides, which play a significant role as ligands for cellular recognition and signaling. In addition, neuronal synaptosomal microdomains are highly enriched with gangliosides, sialidase, and sialyltransferase for dynamic glycocalyx remodeling (Sonnino et al. 2018). It has been shown that the number and positioning of sialic acid residues in these domains modulate calcium influx, the principle second messenger of synaptic activity (Ledeen and Wu 2002; Gleichmann and Mattson 2011).

A previous study from our laboratory explored the N-glycoproteomic profile of the elderly human brain across 11 functional brain regions with age-matched Alzheimer-confirmed subjects (Tena et al. 2022). In this work, we have continued this path of research by comprehensively analyzing the glycosphingolipids of the frontal cortex, temporal cortex, occipital cortex, parietal cortex, cingulate cortex, posterior hippocampus, caudate nucleus, thalamus, lateral cerebellum, and pons using nanoflow MEA Chip Q/ToF mass spectrometry. This methodology quantitatively analyzes these low-abundant lipids to reveal the heterogeneity between regional, age, and disease states. The profiling of these structurally diverse lipids utilized highly sensitive nanoflow chromatography, separation of glycan and ceramide isomers, reproducible chromatographic elution, collision-induced dissociation MS_2_, and accurate mass spectrometric detection.

## Results

**Fig. 1:**
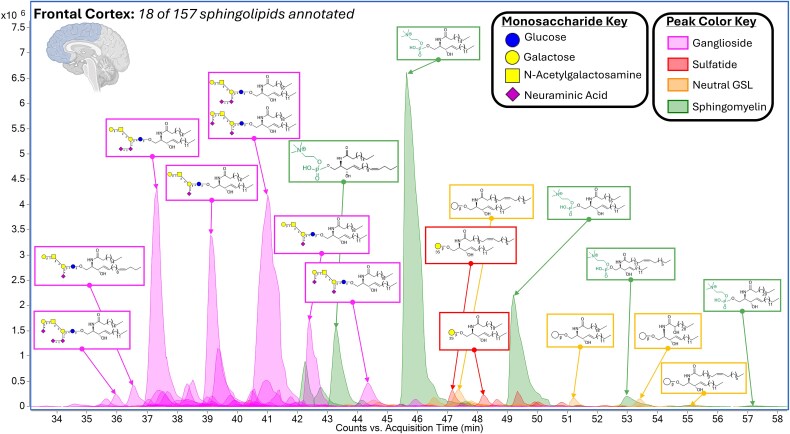
Example chromatogram of human brain tissue by nanoHPLC-MEAchip-Q/ToF analysis. This chromatographic separation, annotating 18 of 157 identified sphingolipids, was used to profile the frontal cortex of a 72-year-old male with no sign of cognitive impairment. Inset structures were assigned by matching biologically relevant molecular structures to accurate mass precursor ions, isotopic ratios, structurally relevant product ions from collision-induced dissociation, and elution patterns.

A total of over 260 intact sphingolipid structures were identified by analyzing the frontal, temporal, parietal, occipital, and cingulate regions of the cerebral cortex as well as the posterior hippocampus, thalamus, caudate nucleus, lateral cerebellum, and pons. Reverse-phase nanoflow Q/ToF accurate mass spectrometry was utilized to quantitatively map the sphingolipid profile of the human brain in the ten different functional regions. The use of biosynthetic pathway knowledge, collision-induced disassociation, and chromatographic retention times to identify compounds was described in detail in a previous publication (Schindler et al. 2024). Briefly, MS^2^ spectra were used to identify molecular structures with fragments corresponding to the headgroup, two-tailed ceramide, and sphingoid base. When comprehensive fragmentation spectra were not obtained, retention time matching of compounds in the same sample and other samples were used to identify low-abundant compounds. Liquid chromatography and ionization used a nanoLC column as part of an MEA Chip (NewOmics, Berkeley) with 1.9-sized particles and post-column solvent adjustment to acquire chromatograms and generate sphingolipid profiles (Fig. 1).

**Fig. 2:**
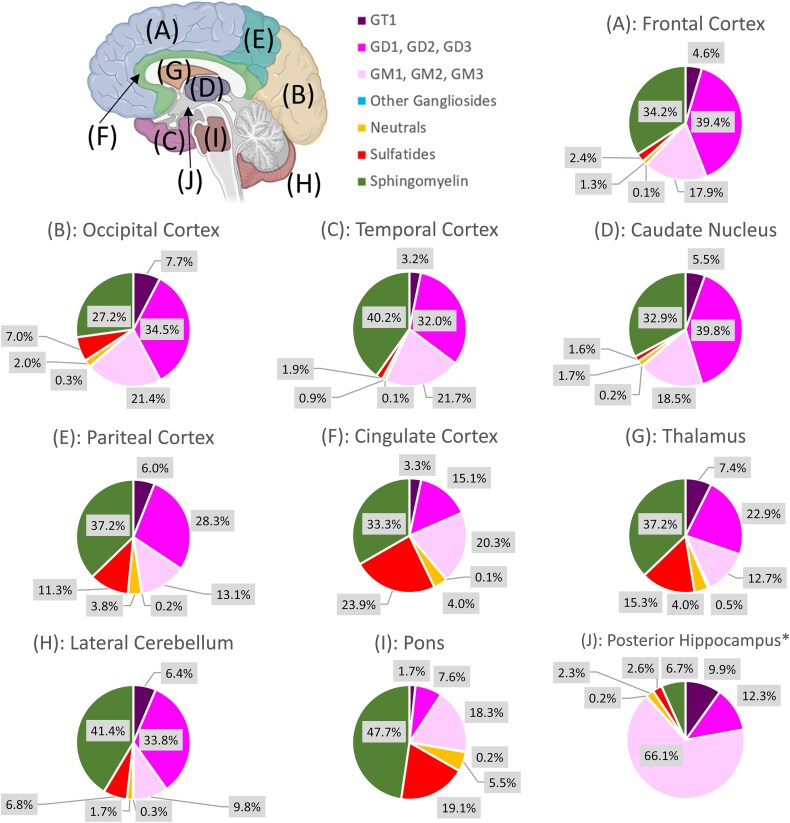
Region specific sphingolipid profile of the major headgroups in ten functional brain regions from relative abundance values obtained from a 72-year-old male with no sign of cognitive impairment. Each headgroup category includes all the observed ceramide structures associated with the depicted headgroups.

Sialic acid-containing glycosphingolipids (GM1_a_, GD1_a&b_, GT1_b_) and sphingomyelin (SM) were the most prevalent headgroups observed in all brain regions (Fig. 2). Additional gangliosides were detected in lower abundances with truncated core structures such as GD2, GD3, GM2, GM3. Also depicted in Fig. 2 are “Other Gangliosides” corresponding to structures detected in trace amounts such as, for example, GalNAc-Fuc-GM1 _a_, Gal-Fuc-GD1, Gal-Fuc-GM1 _a_, Fuc-GD1, Fuc-GM1 _a_. Acetylation and lactonization of sialic acid residues were also observed, adding to the structural diversity. Neutral (GalCer, LacCer) and sulfated GSLs (SM4, SM3) expressed a diverse ceramide profile with long and unsaturated N-linked acyl groups. The major ceramide structures for all brain regions contained a sphingosine base (d18:1, 264.3 m/z) and N-linked acyl groups with 18 and 20 carbons. Minor sphingoid base species included C20-sphingosine (d20:1, 292.3 m/z), dihydroceramide (d18:0, 284.3 m/z), 4-hydroxydihydrosphinganine (t18:0, 300.3 m/z), and 4 t,14c-Sphingediene (d18:2, 262.3 m/z). The N-linked acyl groups were deduced by identifying MS^2^ ions corresponding to the two-tailed ceramide and sphingoid base. The N-linked acyls ranged from 14 to 26 carbon lengths, with hydroxylation typically observed in C24 and saturation in C22 to C26 groups. Odd-numbered carbon acyl groups were also detected in lower abundances. Although this workflow can distinguish and identify different compositions and various isomers, identifying branched lipid isomers would require additional standards or techniques. The comprehensive compound list for all brain regions and subjects has been included (Table S1).

**Fig. 3:**
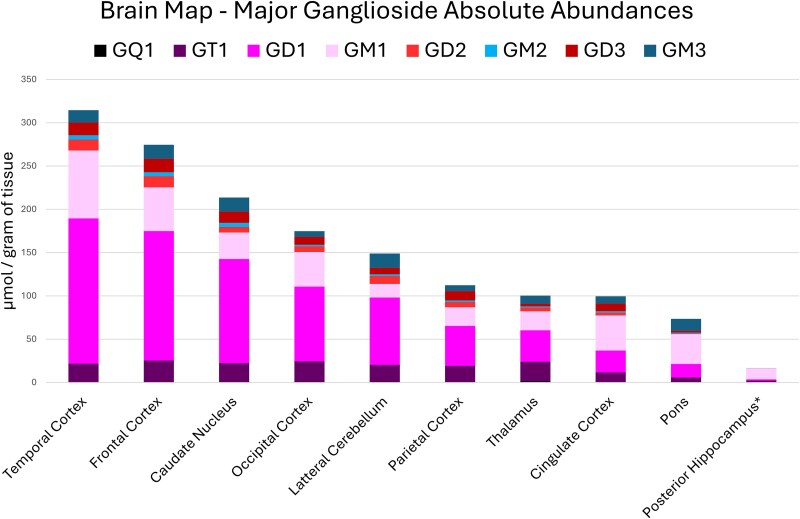
Absolute quantitation of the ganglioside profile in ten functional brain regions from relative abundance values obtained from a 72-year-old male with no sign of cognitive impairment. Each representative ganglioside included both the a- and b-series glycan structures for the major ceramide structures d18:1/C16, C18, C20, and C22 when observed in the depicted region.

Pooled external standards including SM-d18:1/C18, SM-d18:1/C24:1, Glucose-d18:1/C24:1, SGalactose-d18:1/C24:1, GM1_a_-d18:1/C18, GM3-d18:1/C18, GD1_a_-d18:1/C18, and GT1_b_-d18:1/C18 were used at biologically relevant concentrations. The pool was diluted to produce a calibration curve for absolute quantitation of the functional brain regions (Fig. S1). Using the responses generated from these standards, the gangliosides without available standards were interpolated to quantify each region’s major gangliosides (Fig. 3). Although glycan and lipid isomers have variable ionization efficiencies that are structurally dependent, these differences were assumed to be negligible for semi-quantitative estimations, and a single regression from the available standard was used.

The temporal cortex had the highest total ganglioside content (314.5 μmoL of total gangliosides/g of wet tissue), followed by the frontal cortex (274.5 μmoL/g), caudate nucleus (213.6 μmoL/g), occipital cortex (174.7 μmoL/g), lateral cerebellum (148.9 μmoL/g), parietal cortex (112.3 μmoL/g), thalamus (100.4 μmoL/g), cingulate cortex (99.6 μmoL/g), and pons (73.5 μmoL/g). The posterior hippocampus (16.5 μmoL/g) was estimated with an initial tissue weight of 2 mg. Exact measurements could not be performed due to limited sample availability, and the estimate was included for completeness. All other regions were normalized by dilution before analysis with 0.25 mg of tissue per μL of diluent with their measured weights.

**Fig. 4:**
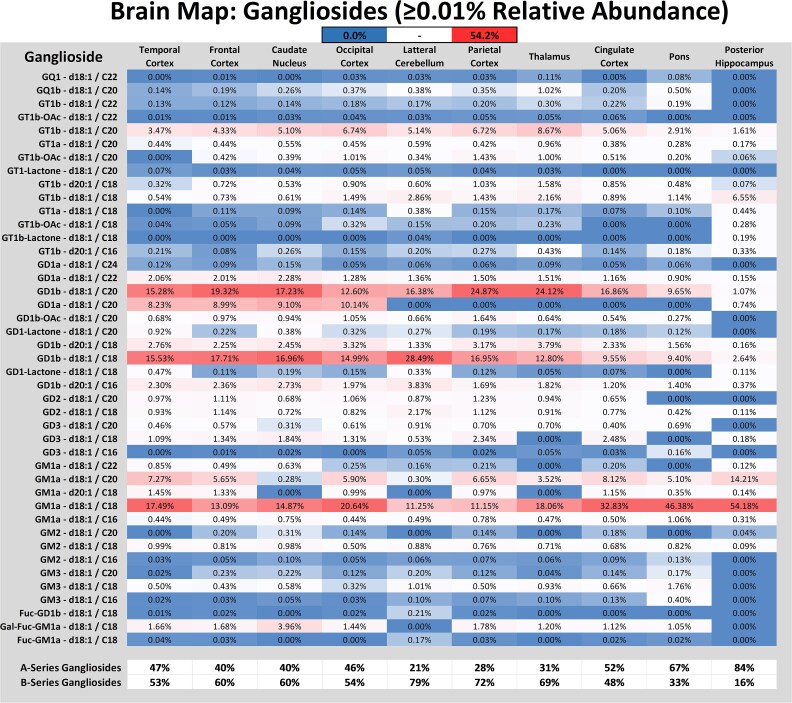
Relative abundance heatmap of all identified intact gangliosides (≥0.01%) with summation of overall a- and b-series structures for ten functional brain regions from a 72-year-old male with no sign of cognitive impairment.

A regional heatmap with the individual intact ganglioside structures (> 0.01% relative abundance) was generated to visualize the resulting profiles (Fig. 4). These relative abundances were used for a nontargeted principal component analysis (PCA), which resulted in two clusters (Fig. S2). Group 1 comprised the frontal cortex, temporal cortex, occipital cortex, and caudate nucleus. These regions generated profiles containing primarily gangliosides (GT1, GD1, and GM1 _a_). Uniquely, they contained GD1_a_-d18:/C20 in ratios comparable to the b-series isomer (1_b_ equal to 0.45–0.8_a_). Group 2 showed more variance between regions but included the parietal cortex, cingulate cortex, thalamus, and lateral cerebellum. GD1_b_-d18:1/C20 was not observed chromatographically for these regions; mass spectral analysis suggests the a-series form is likely present but in much lower abundances. These profiles also contained an increased abundance of sulfatides (SM4, SM3) and neutral glycosphingolipids (Gal, Lac), generating similar overall ceramide profiles. The pons was not grouped in a cluster as its profile was comprised primarily of GM1_a_, SM4, and GalCer, a profile typical in white matter brain tissue. The posterior hippocampus contained mostly GM1_a_ (68.5%) and minor amounts of GD1 and GT1.

**Fig. 5:**
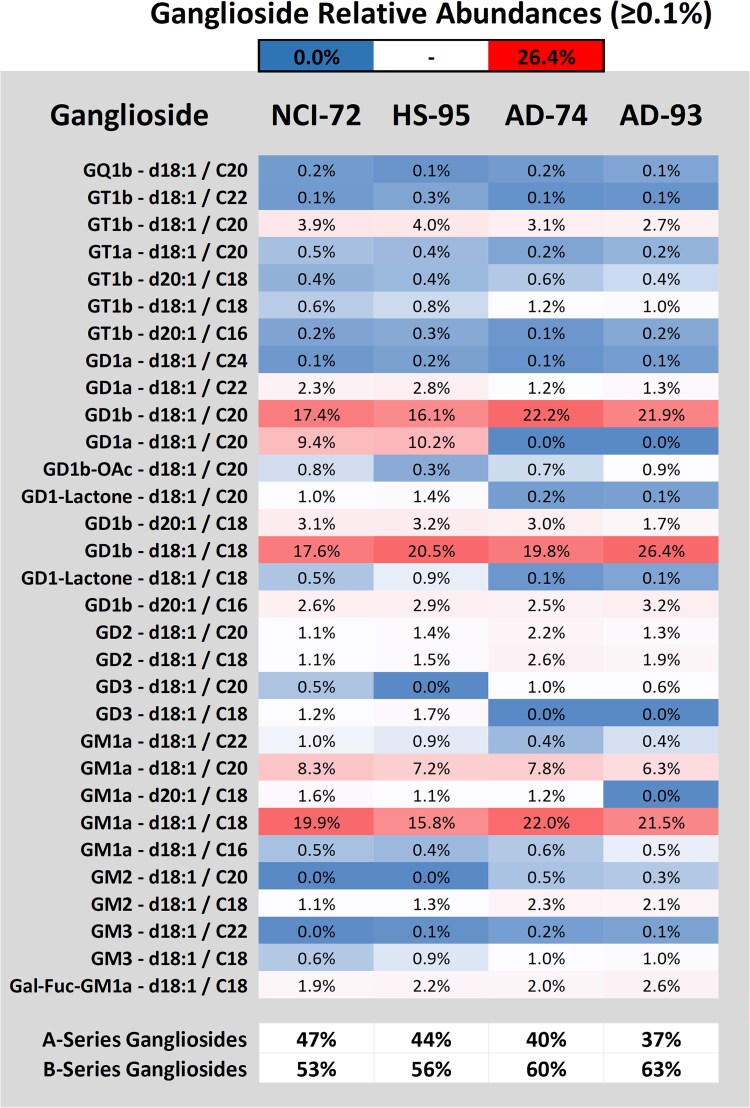
Relative abundances of gangliosides with ≥0.1% observed in the temporal cortex for comparison of age matched controls NCI-72 & HS-95 to autopsy confirmed Alzheimer disease subjects AD-74 & AD-93. Summation of overall a- and b-series structures included all depicted ceramide structures.

**Fig. 6:**
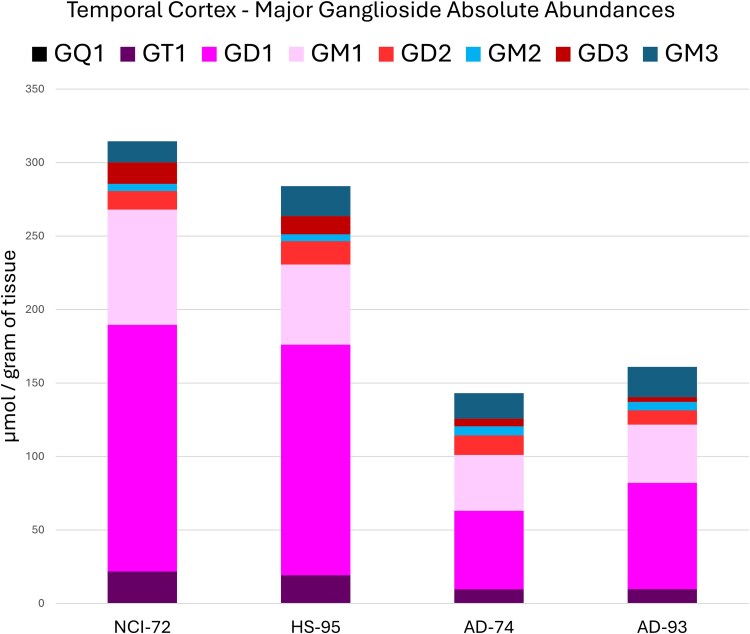
Absolute quantitation of the major ganglioside species observed in the temporal cortex for comparison of age matched controls NCI-72 & HS-95 to autopsy confirmed Alzheimer disease subjects AD-74 & AD-93. Each representative ganglioside included both a- and b-series glycan structures for the major ceramide structures d18:1/C16, C18, C20, and C22.

Glycosphingolipids were profiled for the temporal cortex with three additional subjects for age and age-matched Alzheimer’s disease (AD) comparisons (HS-95, AD-74, AD-93). Using the relative abundance profiles, PCA generated two clusters that resulted in control and AD groupings (Fig. S3). Age comparison of the controls showed minimal changes in the relative glycosphingolipid profile, with a slight increase in GD1, GD2, and fucosylated species and a decrease in GM1_a_ (Fig. 5). Summation of both a- and b-series gangliosides showed a slight shift in the ratio towards b-series gangliosides. Absolute quantitation of age-related changes provided a more complete picture showing an overall decrease (−9.7%) in total gangliosides (Fig. 6). Changes in individual ganglioside abundance due to age were observed by decreases in GQ1 (−42%), GM1_a_ (−31%), and GD3 (−15%). GD2 (+26%) and GM3 (+42%) were found to increase. Comparison of the ganglioside relative abundance profiles of AD subjects to their respective age-matched controls showed the most prominent change to be the absence of GD1a-d18:1/C20 which was apparent after inspection of each sample’s chromatogram and the primary reason for the shift in a- and b-series gangliosides. Additionally, the overall ceramide profile showed a trend of decreasing acyl length where C22 and C20 decreased, and an increase in C18 acyls was observed amongst ganglioside structures (Fig. S4). Absolute quantitation resulted in an overall reduction in gangliosides for both AD-72 and AD-95 (−55% & -43%) primarily due to loss of GD1 (−68% & -54%), GT1 (−56% & -50%), and GD3 (−64% & -73%). GM2 (+27% / +23%) and GM3 (+21% / +1%) were found to increase despite the overall loss of gangliosides.

A notable limitation of this work is the limited number of sample replicates for both regions and ages. This preliminary study profiled ten different human brain regions of a single subject using a single sample per region. Although age-matched regional homogeneity has been observed in human membrane lipids, sampling bias must be considered as <100 mg of a single subject were used to generate the reported sphingolipid profiles. Our goal in this work was to provide data for previously unmapped brain regions and investigate the degree of variability between brain regions, age, and disease state. Our laboratory’s initial work in this study analyzed the N-glycans and N-glycoproteins, which included most regions from the three additional subjects for age (HS-95) and Alzheimer’s disease (AD) comparisons (AD-72 & AD-93) (Tena et al. 2022). Due to sample limitations, this continuation study included the three additional subjects for the temporal cortex only (Table S2).

Additionally, the lack of available standards required interpolating the regression of some abundant gangliosides to provide semi-quantitative abundances. These interpolations considered both the headgroup and ceramide moieties’ effect on the analyte response, but there is still an unknown degree of error associated with these estimations.

## Discussion

In this study, we used nanoflow high-performance liquid chromatography with a C-18 MEA Chip and accurate mass Q/ToF mass spectrometry to quantitatively map the glycosphingolipids of the elderly human brain in a region-specific manner. The subject was a 72-year-old male who showed no cognitive impairment (NCI-72). The ten functional brain regions analyzed were the frontal cortex, temporal cortex, parietal cortex, occipital cortex, cingulate cortex, hippocampus, thalamus, caudate nucleus, lateral cerebellum, and pons. In agreement with the literature, headgroups GM1_a_, GD1_a_, GD1_b_, and GT1_b_ with d18:1/C18 and C20 ceramides were the most abundant glycosphingolipids among all the regions in this study (Sipione et al. 2020).

Like previous studies that mapped ganglioside expression in the human brain, we found the most prominent source of regional heterogeneity was due to differences in the ratios of the major ganglioside species (Kraĉun et al. 1984). An interesting discovery from this study found that the expression of a- and b-series GD1 isomeric gangliosides was correlated to the ceramide’s acyl group. Longer chain acyl groups (C24 & C22) were identified as GD1_a,_ while shorter chain acyls (C16 & C18) were found to be the GD1_b_ isoform. As mentioned in the results, d18:1/C20 species were predominantly the b-series ganglioside for all regions. Still, they showed a high abundance of GD1_a_ in the frontal cortex, temporal cortex (NCI-72 & HS-95), occipital cortex, and caudate nucleus regions commonly associated with integrated sensory processing, memory, and motor control (Micah M. Murray and Mark T. Wallace 2012). It’s unclear if this structural specificity occurs in the Golgi during glycan synthesis, vesicle transportation, after outer membrane incorporation, or in combination. It was reported that *V. Cholerae* sialidase preferentially hydrolyzes the terminal sialic acid of GD1a with shorter lipid chains in vesicles. Further, the sialidase activity was increased by 1.5 to 3-fold in the presence of Ca2+ ions in vesicular and micellar dispersions (Masserini and Tettamanti 1988). Previous works have also shown sialic acid enzyme activity in synaptosomal microdomains, which dynamically adjust the local ganglioside profile (Tettamanti et al. 1972; Preti et al. 1980). The observed correlation between glycan and ceramide structure, we hypothesize, is likely due to the positioning of the lipid tail in a cellular membrane, which has a steric effect on sialidase activity. The mechanism to desiaylate gangliosides favors the hydrolysis of terminal Neu5Ac residues over those at the membrane interface. This also has significant implications on lipid raft organization, where full GT1_b_ and GM3 will show physiological differences in shaping their surroundings. The specificity of the interactions with membrane proteins typically depends on the number and positioning of sialic acid residues.

The standards employed in this study were chosen to determine the response for the most abundant headgroup and ceramide structures in the human brain. The regression varied due to both moieties, so interpolations were made to estimate the response for standards that were not available. There are limited studies that can be directly compared to our results as comprehensive analysis of intact glycosphingolipids for specific age groups and brain regions is rare, and the available data shows high variability in the expression of gangliosides for different regions and developmental stages. Additionally, quantitative results vary based on normalization techniques to total ion count, lipids, proteins, or tissue mass. Svennerholm et al. previously reported quantitative information on total gangliosides in the frontal and temporal cortex for subjects aged 70 and 90 (Svennerholm et al. 1994). After assigning matching units to our quantitative analysis, similar conclusions were drawn. Our quantitative results for total gangliosides in the frontal cortex in the 70-year age range (3.18 μmol bound Neu5Ac/g of tissue), the temporal cortex for 70 (3.45 μmol Neu5Ac/g), and temporal cortex for 90 (3.18 μmol Neu5Ac/g) were within the biological standard deviation reported in the literature. Similarly, the ratios of individual gangliosides showed consistent trends, with GD1 being the dominant species, followed by GT1, GM1_a_, and the minor gangliosides. The temporal cortex age comparison of control subjects from 70 to 90 years conferred with previous studies showing a minor decrease in total abundance of gangliosides (−8.7%), an increase in GM3, and a shift towards b-series gangliosides. The differences in our results can likely be attributed to the differences in methodologies and sampling limitations. Our results from absolute quantitation of total gangliosides produced similar regional trends with a more comprehensive study mapping the bound Neu5Ac content in the human brain (Kraĉun et al. 1984). However, quantitative results cannot be directly compared as the results from the literature are normalized to the total protein content.

Alzheimer’s disease (AD) is the most common form of neurodegeneration observed in humans, which affects the person’s memory, cognition, and behavior. The progressive disorder causes brain shrinkage due to neuronal cell death, and a hallmark symptom is an accumulation of both amyloid-β (Aβ) plaques and Tau tangles. Despite significant resources allocated to understanding this disease, the neuropathological mechanisms are still not fully understood. Gangliosides have been indicated to play a role in AD pathology in numerous instances(Ariga, McDonald, and Yu 2008). Previous work by Kracun et al. showed a significant decrease of GT1b, GD1b, GD1a, and GM1 _a_ in the frontal and temporal cortex of AD-affected brains measured in lipid-bound sialic acid (Kracun et al. 1992). Our results confirm these findings for the temporal cortex and provide additional information on the ceramide backbone in the changing ganglioside expression. Our observation of GD1a-d18:1/C20 loss in the temporal cortex compared to age-matched controls suggests that neurons expressing this ganglioside are lost in the progression of AD neurodegeneration. A study using GD1a monoclonal antibodies found this ganglioside was enriched on dystrophic neurites with Aβ plaque, suggesting the ganglioside may play a role in the aggregation of these plaques and the disease pathology leading to neuronal death, explaining their absence in AD subjects (Nishinaka et al. 1993). An additional explanation for the loss of GD1a is due to microglial activation upon inflammatory stimuli, resulting in the secretion of neuraminidase-3 active vesicles (Delaveris et al. 2023).

We also observed that GM1_a_ constituted a larger fraction of the total ganglioside profile in AD subjects. A well-studied interaction involves soluble Aβ to the insoluble β-sheet structure through direct binding with clustered GM1_a_ gangliosides and cholesterol. Insoluble Aβ subsequently acts as a seed for continuous amyloid plaque formation on neuronal synapses.(Yanagisawa 2015) Interestingly, GM1_a_ clustering is enhanced by increased cholesterol in the lipid environment (Arumugam et al. 2021), and over-accumulation of cholesterol has been reported in AD brains (Feringa and van der Kant 2021). Cholesterol is tightly regulated by apolipoprotein E (APOE), and the APOE4 ε4 genotype is a genetic marker associated with increased AD risk.

Transcriptomic analysis of AD brains shows an upregulation of ceramide synthase 1 and 2 (CerS1 & CerS2), which are responsible for the addition of C18 only and C18, C20, and C22 acyl groups, respectively (Czubowicz et al. 2019). However, Couttas et al. reported that CerS2 shows loss of activity in the temporal cortex as early as Braak stage I despite upregulation (Couttas et al. 2016). Our results supported this conclusion with decreases in ganglioside-associated C20 and C22 acyls and an increase in C18 for AD-73 (+6.6%) and AD-95 (+9.2%). Whether the changes observed in the ceramide profile are directly correlated to changes in the ganglioside’s glycan structure is unclear, but differences in lateral organization and interdigitation are likely occurring.

## Conclusion

A region-specific analysis of the human brain’s glycosphingolipid profile was conducted using nanoflow MEA Chip Q/ToF mass spectrometry. This study found most of the heterogeneity between regions was in ganglioside structures. From the methods ability to separate glycan and lipid isomers, unique identifying product ions, and highly sensitive nanoflow LC–MS, we were able to provide insights to the region specific intact glycosphingolipid profile of the aged human. Further, we found a correlation between ceramide and ganglioside structure, where acyl length affected whether an a-series or b-series ganglioside was expressed. Future studies will benefit from a larger sample set with a targeted sampling of neuronal synapses. Additional assays for membrane cholesterol, phosphatidylserine, and proteins would further elucidate the environment of the bioactive synaptosomal landscape.

## Materials and methods

### Materials and chemicals

Sphingomyelin-d18:1/C18 (Cat# 860586), Sphingomyelin-d18:1/C24:1 (Cat# 860593), Glucose-d18:1/C24:1 (Cat# 860549), SM4-d18:1/C24:1 (Cat# 860571), GM1a-d18:1/C18 (Cat# 860588), GM3-d18:1/C18 (Cat# 860074), GD1a-d18:1/C18 (Cat# 860091), and GT1b-d18:1/C18 (Cat# 860089) standards were purchased from Avanti Polar Lipids (Alabaster, AL). Lymphoblast CESS cells (Cat# TIB-190) were obtained from the American Type Cell Culture (Manassas, VA). Human serum (Cat# S7023), sucrose (Cat# S7903), KOH (Cat# P5958), ammonium acetate (NH_4_CH_3_CO_2_, Cat# 73594), sodium carbonate (Cat# S5761), and protease inhibitor cocktail (Cat# 539137) were purchased from Sigma (St. Louis, MO). Fetal bovine serum (Cat# 16000–069), penicillin–streptomycin (Cat# 15140–122), 1 M HEPES (Cat# 15630080), methanol (MeOH, Optima LC/MS, Cat# A456–4), and isopropanol (IPA, Optima LC/MS, Cat# A461–4) were purchased from ThermoFisher Scientific (Waltham, MA). C-8 SPE plate (100 mg, Cat# FNSC08.800) was purchased from Glygen. Glacial acetic acid (GAA, Cat# AC110) was purchased from Spectrum (New Brunswick, NJ). Formic acid (Optima LC/MS, Cat# A117–50) was purchased from Fisher Chemical (Hampton, NH).

### Brain tissue

Human brain tissue was obtained through the University of California, Davis – Alzheimer’s Disease Center from donors’ postmortem and stored at −80 °C before processing and analysis. Four different brains were profiled; the first showed normal cognitive function and was considered the control sample (**A**, NCF-72). The second had hippocampal sclerosis (**B**, HS-95). The last two subjects were age-matched with autopsy-confirmed Alzheimer’s disease (**C**, AD-74 & **D**, AD-93). Up to ten brain regions were collected from each donor: frontal cortex, temporal cortex, parietal cortex, occipital cortex, cingulate cortex, hippocampus, thalamus, caudate nucleus, lateral cerebellum, and pons.

### Standard preparation

External standards SM-d18:1/C18, SM-d18:1/C24:1, Glc-d18:1/C24:1, SHex-d18:1/C24:1, and GM1a-d18:1/C18 were received as ammonium salts which were weighed and diluted to 50 μM stock solutions in MeOH/IPA/water (2/8/1, v/v/v%). Further dilutions used MeOH/water (1/1, v/v%). GM3-d18:1/C18 (100 μg/mL), GD1a-d18:1/C18 (100 μg/mL), and GT1b-d18:1/C18 (124 μg/mL) were received as MeOH solutions, and diluted in MeOH/water (1/1, v/v%). To minimize any hygroscopic absorption of atmospheric water, all standards were allowed to acclimate to room temperature before subsequent solution preparation. Standard curve solutions were processed with the same procedure as samples to minimize bias associated with the sample preparation.

### Sample preparation

The sample profiles were generated with 10-100 mg of human brain tissue. Tissues were individually weighed into 15 mL falcon tubes and diluted with 1.5 mL buffer of 0.25 M sucrose, 20 mM HEPES adjusted to pH 7.4 with KOH, and a 1:100 protease inhibitor cocktail. Homogenization was first accomplished manually, then by 1–2 sequences of lysis with μ-needle sonication with a maximum of 80 J/sequence until a fine suspension was obtained.

The nuclear fraction was precipitated by centrifugation at 2000 RCF for 10 minutes. The transparent supernatant was transferred and ultracentrifuged at 200 k RCF for 30 minutes at 4 °C to form a membrane pellet. After removing the supernatant, samples were diluted with 1 mL of 0.2 M sodium carbonate and ultracentrifuged to remove membrane-associated proteins. The supernatant was removed, and samples were ultracentrifuged again with the same volume of water. After discarding the water, membrane lipids were dissolved using a modified Folch extraction using 800 μL of freshly prepared water/MeOH /CHCl_3_ (3/8/4, v/v/v%) and sonicated at RT for 30 min. Samples were then centrifuged at 9000RCF for 10 min to precipitate the membrane proteins, and the supernatant was transferred to a fresh vial. 100 μL of 0.1 M KCl was added to the transferred supernatant, inducing a liquid–liquid separation. The top layer (aqueous) containing phospholipids and sphingolipids was transferred and dried by vacuum centrifugation. This modified Folch extraction enriches for all polar lipid structures with a higher preference toward highly polar and ionized species; sphingolipids (1-OH ceramides (Cer-OH), sphingomyelin (SM), cerebrosides (GalCer & GlcCer), sulfatides (SM4 & SM3), neutral complex GSLs, anionic complex GSLs & glycerophospholipids (phosphatidylethanolamine (PE), phosphatidylcholine (PC), phosphatidylinositol (PI), phosphatidylserine (PS), and cardiolipin (CL)). Recoveries of individual structures depend on the structure of both the headgroup and lipid tail. Therefore, some species, such as very long lipid chain cerebrosides, may be under-represented when using relative abundances.

Sphingolipids were enriched with a 100 mg, C-8, 96-well SPE plate. Wells were first washed with 1200 μL of MeOH/IPA (1/1, v/v%) and primed with 400 μL of water/MeOH (1/1, v/v%). Samples were reconstituted with 600 μL of water/MeOH and gravity loaded; the flow through was reloaded to ensure maximum recovery. Sample wells were washed with a total of 1.2 mL of water/MeOH. Sphingolipids were eluted with 600 μL of the MeOH/IPA and then dried. The condition, prime, wash, and elution steps were performed using centrifugation (100 RCF, 1 minute).

Dried samples were sealed and stored at −80 °C until ready for analysis. Before analysis, samples were reconstituted to 0.25 mg of tissue/μL with water/MeOH (1/1, v/v%), transferred to autosampler vials, and stored in the autosampler operating at 4 °C before injection.

**Fig. 7:**
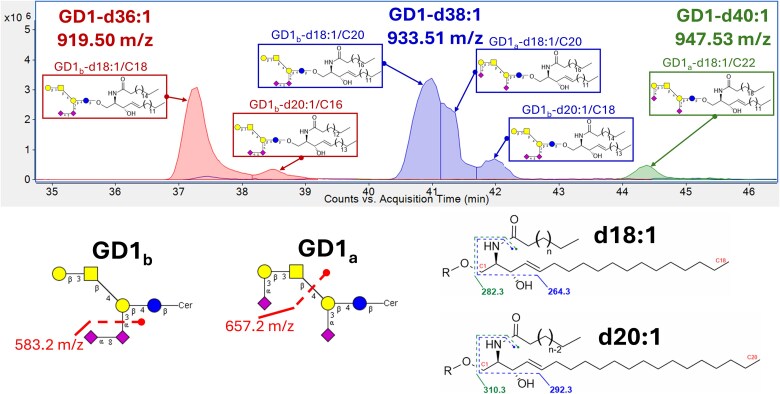
Example ganglioside elution profile and identifying MS^2^ product ions produced by nanoHPLC-MEAchip-Q/ToF analysis. Depicted isomeric structures include glycan a- & b-series isomers (GD1a & GD1b) with ceramide isomers (d18:1/CN & d20:1/CN).

### Nanoflow MEAChip Q/TOF methodology

Automated sample injection and data collection used an Agilent 1200 series nanoflow HPLC. On-line sample enrichment used a Zorbax 300SB-C8 trap column, 0.3 ID x 5 mm, 5 μm particle size, 300 Å pore size (Agilent Technologies Inc., Cat# 5065–9914). The chromatographic separation and analyte ionization were performed using a C-18 MEAChip, 1x 10 μm nozzle, 0.07 ID x 150 mm, 1.9 μm particle size (NewOmics, Cat# C1005). The loading/washing pump was operated at 2.5 μL/min. Sample loading used 0.1% GAA and 20 mM NH_4_CH_3_CO_2_ in water/MeOH/IPA (40/50/10, v/v/v%). Sample washing (MP-W) used MeOH/IPA (1/1, v/v%). The gradient was as follows: 0% MP-W from 0 to 20 min, increased to 95% at 25 min, held until 35 min, decreased to 0% at 40 min, and held until 70 min. The analytical gradient used a flow rate of 0.25 μL/min. Mobile phase A (MP-A) used 0.1% GAA in 20 mM NH_4_CH_3_CO_2_in MeOH/water (25/75, v/v), and mobile phase B (MP-B) with a composition of 0.1% GAA in 20 mM NH_4_CH_3_CO_2_ in MeOH/IPA (75/25, v/v). The timed composition changes are as follows: 85% MPB from 0 to 15 min, a linear increase to 100% at 40 min, held until 55 min, decreased to 85% by 60 min, and held until 80 min. The C-8 trap column was operated at 70 °C. A 10 pt/2 ps μ-switching valve was configured for efficient enrichment, analyte transfer, and washing at low flow rates. Samples are enriched from 0 to 6 min (μ-valve 1 → 10). From 6 to 20 min, analytes are backflushed from the C-8 trap to the MEAChip source (μ-valve 1 → 2). From 20 to 80 min, the gradient, wash, and re-equilibration are carried out (μ-valve 1 → 10).

The MEAChip source was operated in positive ion mode, and the solvent composition was adjusted through the post-column inlet (PCI) with a flow rate of 0.25 μL/min. The PCI solution was made freshly in 1 mL preparations with a composition of formic acid/water/MeOH (5/2.5/92.5, v/v/v%) and spiked with 1 μL of a 200,000x diluted 1221.9 m/z correction mass in MeOH (Agilent Technologies Inc., Cat# G1982–85001). Precursor ion mass filtering, fragmentation, and detection were performed on a quadrupole time-of-flight mass spectrometer (Agilent Technologies, G6520A). Source conditions were optimized by direct infusion and using N_2_ drying gas at 250 °C with a flow rate of 2.0 L/min. The capillary voltage was 3200 V for a corresponding capillary and chamber current of 0.165 μA and 2.20 μA, respectively. The fragmentor, skimmer, and octopoleRF voltages were set to 175 V, 90 V, and 750 V, respectively. The quadrupole used automatic precursor ion selection with a mass range of 575–2000 m/z and an absolute threshold of 10,000 counts with a maximum of 5 precursors per cycle and narrow isolation width (~1.3 amu). The preferred charge state was set to 2 > 1. Precursor ions were fragmented in an N_2_-filled chamber with collision-induced disassociation using an m/z dependent collision energy determined by linear interpolation with the equation $E=1.2\times \left(\frac{{m}\left/ {z}\right.}{100}\right)+12$. Active exclusion was enabled after collection of one MS_2_ spectrum and released after one-minute corresponding to approximately one half the average peak width. The time-of-flight detector was operated such that abundance and accurate mass were in the range of 100–2000 m/z.

### Data analysis

Post-acquisition compound identification and peak integration were completed using Agilent’s MassHunter Qualitative Analysis software version (B10.00) with the Find by Molecular Feature (FMF) algorithm using a CSV database of compounds, including the molecular formula, mass, name, and description. Each sample’s compound list was exported to individual CSV files. An in-house Python script was used to organize the data into a single spreadsheet for analysis in Microsoft Excel and GraphPad Prism.

### Absolute quantitation

11-point calibration curves were generated with external standards comprising the major headgroups and ceramide structures. However, not all major species had an external standard available, so interpolations were required, which accounted for both the glycan headgroup and ceramide backbone’s effect on ionizability. The responses of SM-d18:1/C18 and SM-d18:1/C24:1 were used to calculate the expected response based on the ceramide tail. Next, the response for each headgroup with a standard available was normalized to the SM response of the same ceramide structure (d18:1/C18 or d18:1/C24:1) to find the change in response based on the headgroup compared to SM. There was no GD3 standard available, so the response was estimated using the GM3 response with the addition of sialic acid. GM2 and GD2 were estimated by the average response of GM1_a_/GM3 and GD1/GD3. With interpolated slopes for GT1, GD1, GM1, GD2, GD3, GM2, GM3 with ceramides d18:1/C16, C18, C20, and C22 absolute abundances were calculated. After quantitation of each species, the headgroups were summed to calculate the absolute abundance of total gangliosides in μmol per gram of tissue for each sample.

### Isomer identification

Isomeric compounds are indistinguishable by accurate mass, isotopic distributions, typically used for identification of compounds with high-resolution mass spectrometry. In this work, we used chromatographic separation and unique product ions to differentiate the various isomeric species such as a-series and b-series gangliosides as well as d20:1 ceramide species. GD1b glycans produced an MS_2_ ion 583.2 m/z corresponding to Neu5Ac-Neu5Ac. GD1a and GT1b generated the ion 657.2 m/z corresponding to the terminal Neu5Ac-Gal-GalNac. GT1a produced a 948.3 m/z ion in high abundance corresponding to Neu5Ac-Neu5Ac-Gal-GalNac. The glycan isomer elution order was also confirmed by spiking with standards and retention time matching. A lipid isomer, C20 sphingosine (d20:1), was also observed for the major gangliosides and was identified by MS_2_ with a 292.3 m/z fragment. Example chromatograms with identifying fragments are included for GD1 (Fig. 7) and GT1 (Fig. S5).

## Supplementary Material

250326_Sup_Info_Final_cwaf022

## Data Availability

The data that support this study’s findings and acquisition report for all instrumental parameters are available in the GlycoPOST mass spectrometry data repository for glycomics (ID# GPST000446).
