# Supplementary material for: Region-specific quantitation of glycosphingolipids in the elderly human brain with Nanoflow MEA Chip Q/ToF mass spectrometry
Source: Glycobiology. 2025 Apr 10;35(6):cwaf022. doi: 10.1093/glycob/cwaf022 (PMC12021261; doi:10.1093/glycob/cwaf022)
Supplement: 250326_Sup_Info_Final_cwaf022 [file 250326_sup_info_final_cwaf022.docx]

**Region-specific Quantitation of Glycosphingolipids in the Elderly Human Brain with Nanoflow MEA Chip Q/ToF Mass Spectrometry**

Key words: Brain Map / Glycosphingolipids / MEA Chip / Nanoflow HPLC-Q/ToF

Ryan L. Schindler^1^, Lee-way Jin^2^, Angela M. Zivkovic^3^, Yiyun Liu^1^, Carlito B. Lebrilla^1^*

^1^Department of Chemistry, University of California, Davis, California, USA.

^2^University of California Davis Medical Center, Department of Pathology and Laboratory Medicine, University of California Davis Medical Center, Sacramento, California, 95817, USA. ^3^Department of Nutrition, University of California, Davis, California, 95616, USA.

*Corresponding Author:
E-mail: [cblebrilla@ucdavis.edu](mailto:cblebrilla@ucdavis.edu).

Supplementary Information:
Figures S1 – S5
Tables S1 - S2


Figure S1.
Calibration curve using pooled external standards for absolute quantitation.

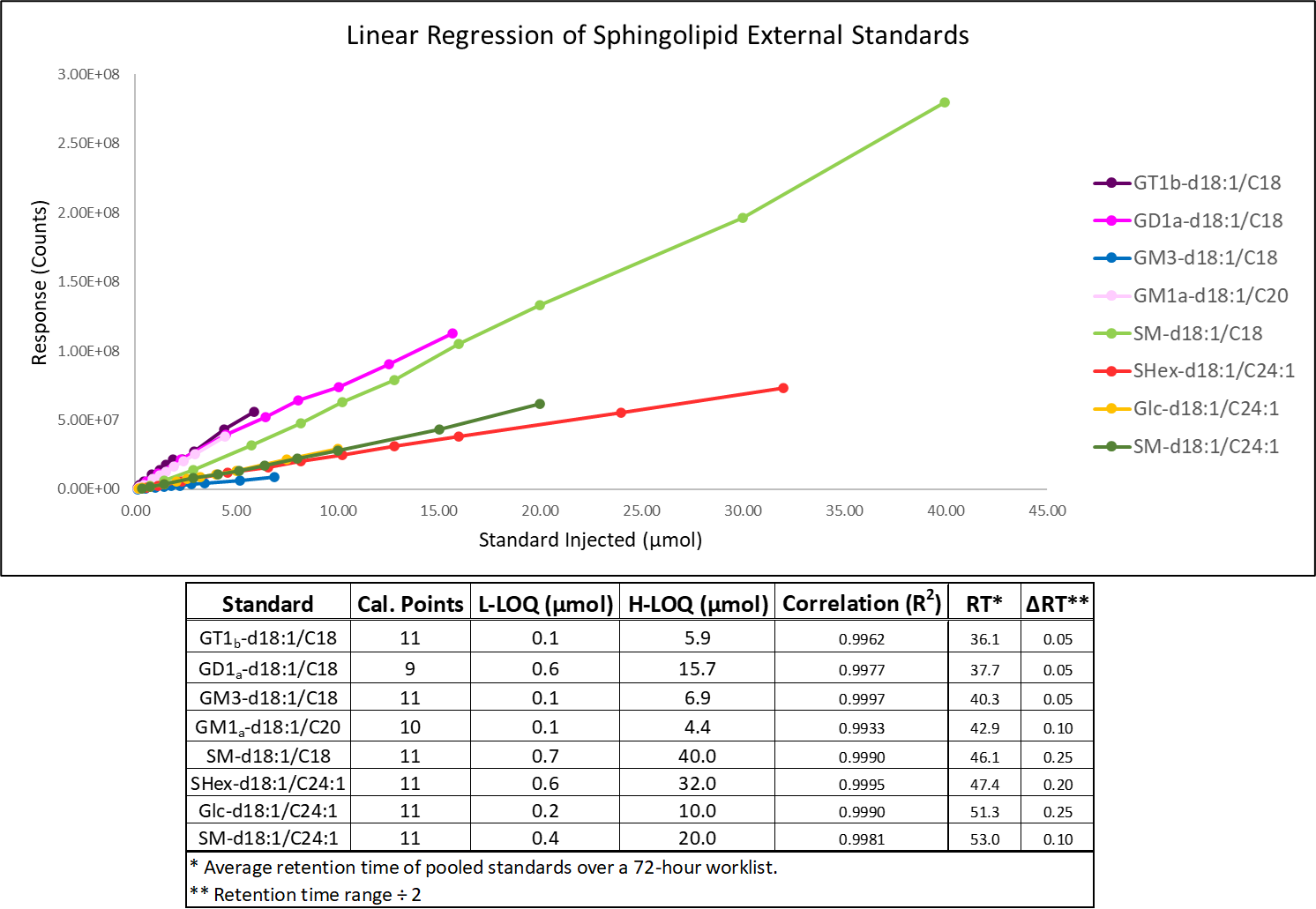


Figure S2.
Principal component analysis of all 10 brain regions resulted in two clusters. Cluster 1 (green) included the frontal cortex, temporal cortex, occipital cortex, and caudate nucleus and cluster 2 (red) included the parietal cortex, cingulate cortex, thalamus, and lateral cerebellum. The posterior hippocampus and pons showed uniqueness in their variance and were not clustered with any other regions.

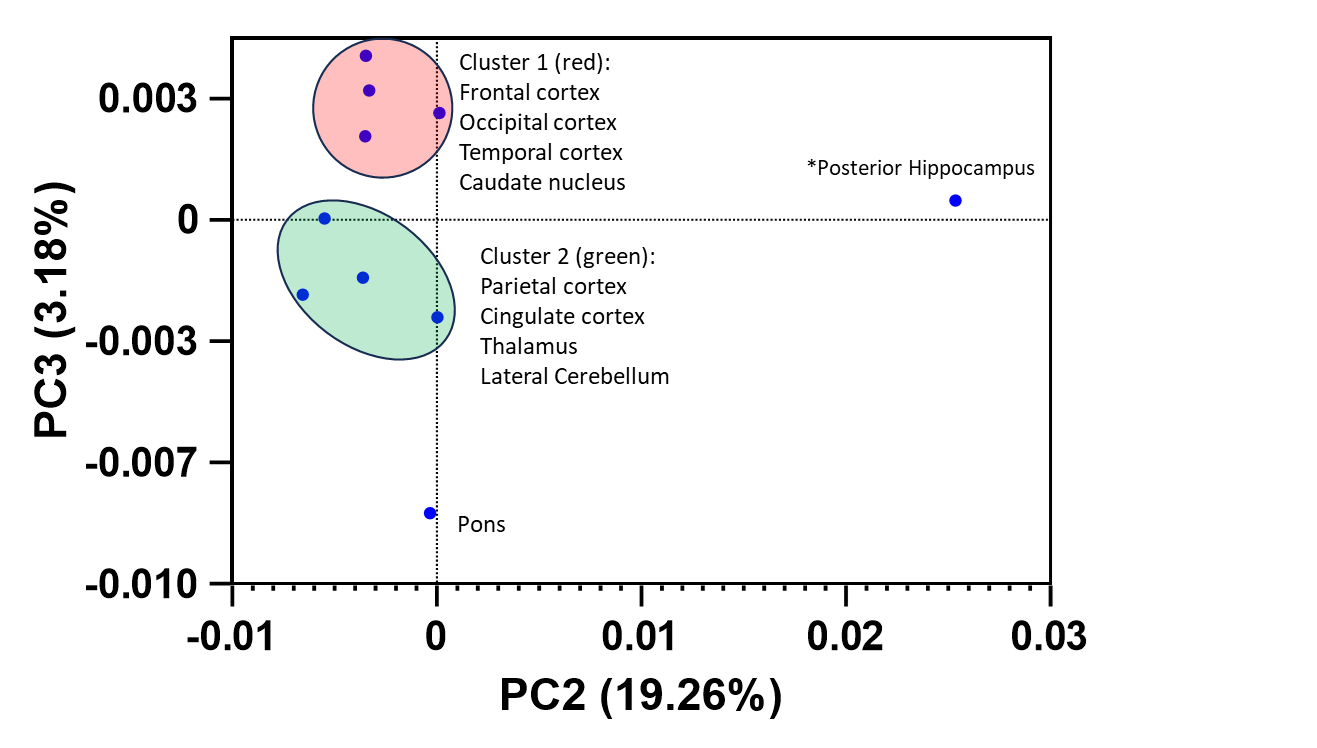

Figure S3.
Principal component analysis of all detected sphingolipids for the temporal cortex of NCI-72, HS-95, AD-74, and AD-93.

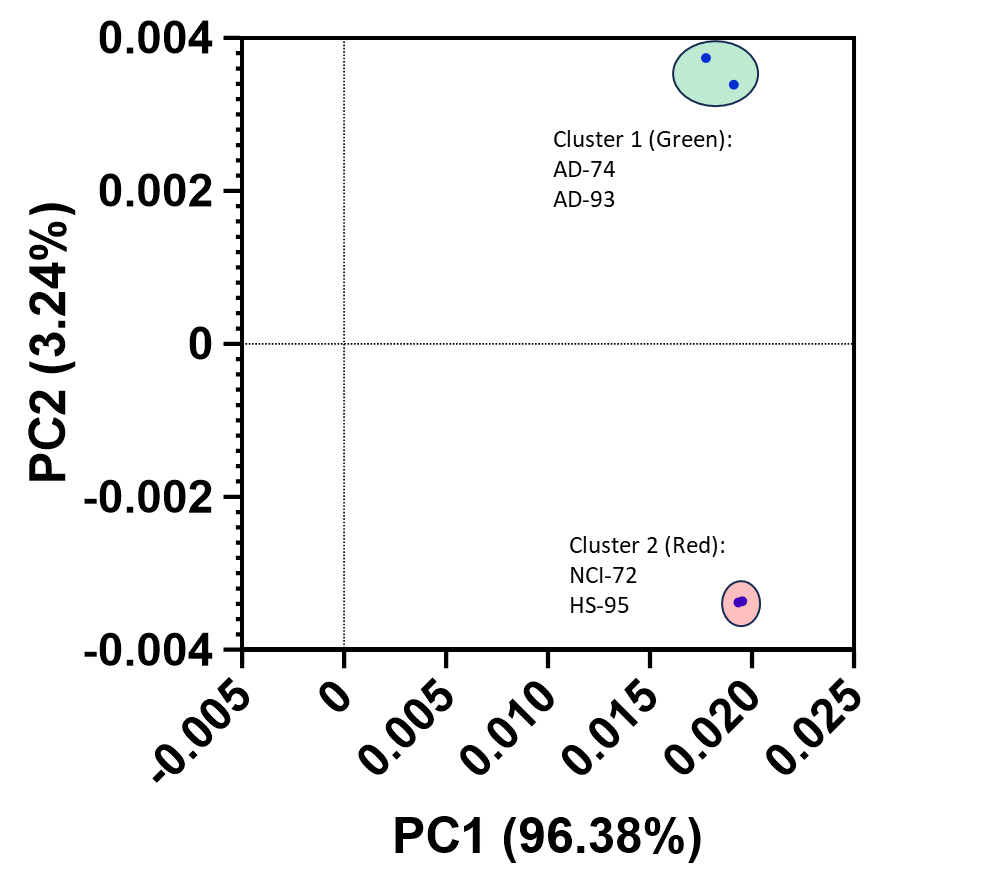


Figure S4.
Relative abundance profile of ganglioside associated ceramide structures for the temporal cortex of NCI-72, HS-95, AD-74, and AD-93.

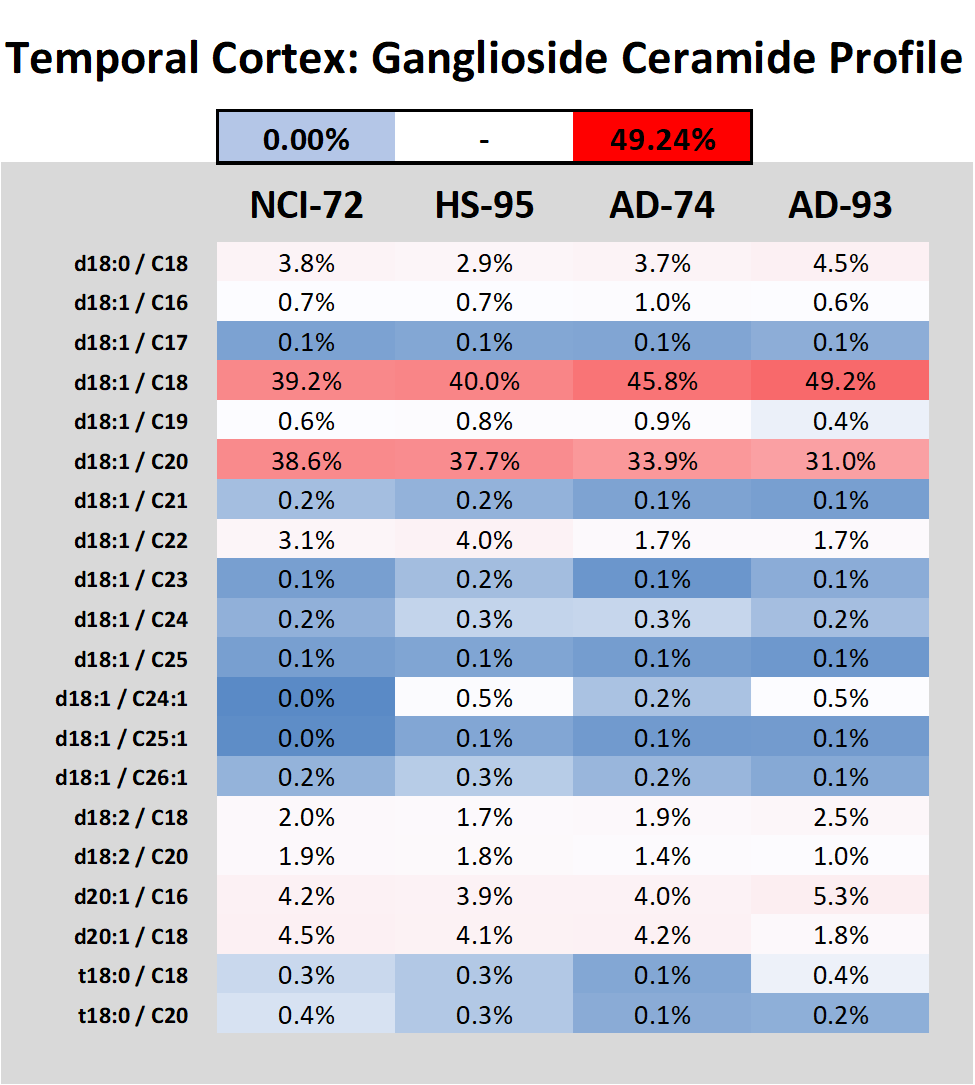


Figure S5.
Elution profile and corresponding fragment ions for identification of ganglioside glycan and lipid isomers: GT1a, GT1b, d18:1/CN, and d20:1/CN.

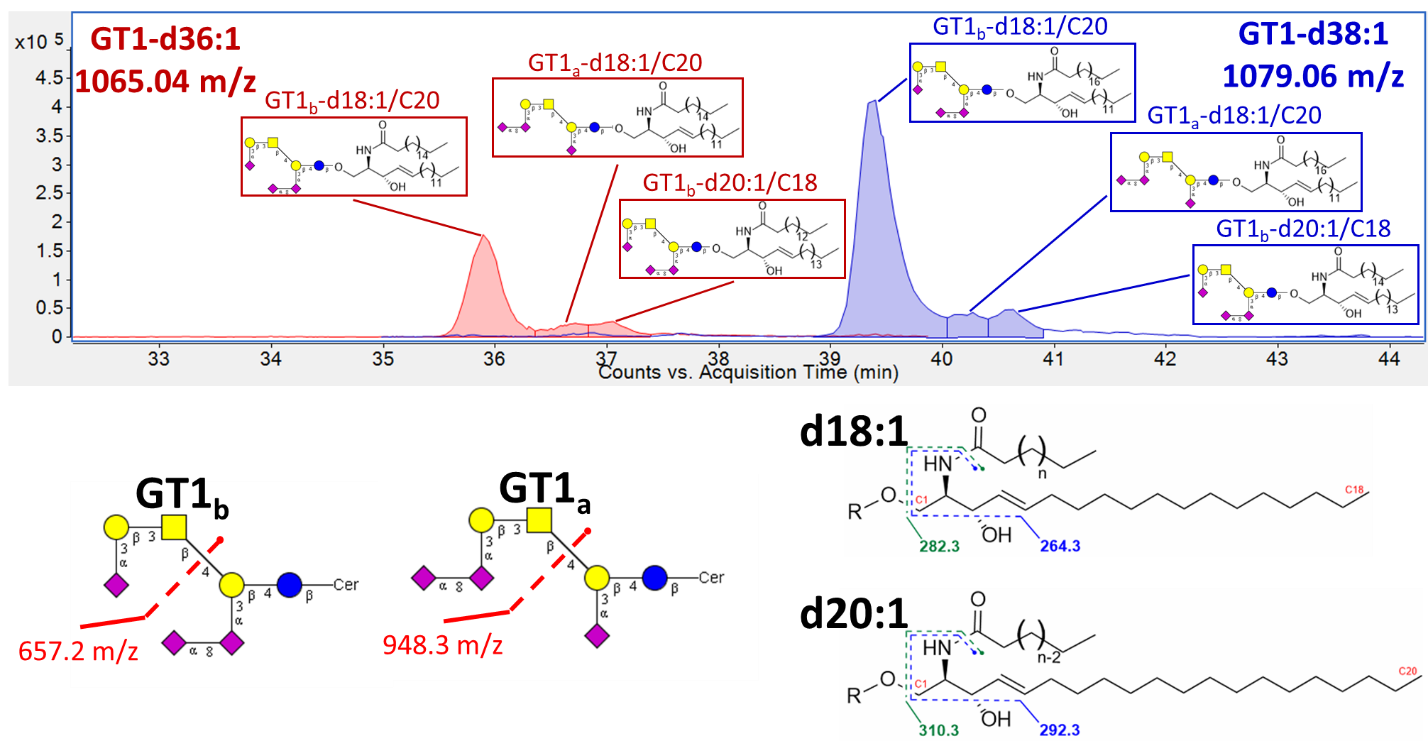


Table S1.
Relative abundances of all identified sphingolipids from regional and subject comparison of the human brain from Chapter IV. NCI-72 regional map of the frontal cortex (A), parietal cortex (B), occipital cortex (C), posterior hippocampus (D), thalamus (E), caudate nucleus (F), Lateral cerebellum (G), pons (H), cingulate cortex (I), temporal cortex (J). HS-95 temporal cortex (K), AD-74 temporal cortex (L), and AD-93 temporal cortex (M).

| Sample: | A | B | C | D | E | F | G | H | I | J | K | L | M |
| --- | --- | --- | --- | --- | --- | --- | --- | --- | --- | --- | --- | --- | --- |
| Total GSLs: | 131 | 131 | 130 | 66 | 131 | 136 | 137 | 120 | 130 | 140 | 146 | 133 | 127 |
| GP1 - d18:1 / C22 | 0.01% | 0.00% | 0.00% | 0.00% | 0.00% | 0.00% | 0.01% | 0.00% | 0.00% | 0.00% | 0.00% | 0.00% | 0.00% |
| GQ1b - d18:1 / C22 | 0.01% | 0.02% | 0.03% | 0.00% | 0.07% | 0.00% | 0.02% | 0.04% | 0.00% | 0.00% | 0.00% | 0.00% | 0.00% |
| GQ1b - d18:2 / C20 | 0.00% | 0.00% | 0.00% | 0.00% | 0.01% | 0.00% | 0.00% | 0.00% | 0.00% | 0.00% | 0.00% | 0.00% | 0.00% |
| GQ1b - d18:1 / C20 | 0.18% | 0.26% | 0.32% | 0.00% | 0.72% | 0.26% | 0.33% | 0.26% | 0.12% | 0.13% | 0.09% | 0.13% | 0.11% |
| GQ1b - d18:1 / C18 | 0.00% | 0.00% | 0.00% | 0.20% | 0.06% | 0.01% | 0.07% | 0.04% | 0.00% | 0.00% | 0.01% | 0.00% | 0.02% |
| GQ1b - d18:0 / C18 | 0.00% | 0.00% | 0.00% | 0.00% | 0.00% | 0.00% | 0.01% | 0.00% | 0.00% | 0.00% | 0.00% | 0.00% | 0.00% |
| GT1b - d18:1 / C26:1 | 0.00% | 0.01% | 0.00% | 0.00% | 0.02% | 0.02% | 0.00% | 0.00% | 0.00% | 0.01% | 0.02% | 0.01% | 0.01% |
| GT1b - d18:1 / C26 | 0.00% | 0.00% | 0.00% | 0.00% | 0.00% | 0.00% | 0.00% | 0.00% | 0.00% | 0.00% | 0.00% | 0.00% | 0.00% |
| GT1b - d18:1 / C25:1 | 0.01% | 0.00% | 0.00% | 0.00% | 0.01% | 0.02% | 0.00% | 0.01% | 0.01% | 0.00% | 0.02% | 0.00% | 0.00% |
| GT1b - d18:1 / C25 | 0.01% | 0.00% | 0.00% | 0.00% | 0.00% | 0.02% | 0.00% | 0.00% | 0.00% | 0.01% | 0.02% | 0.00% | 0.00% |
| GT1b - d18:1 / C24:1 | 0.00% | 0.00% | 0.00% | 0.19% | 0.00% | 0.02% | 0.00% | 0.00% | 0.02% | 0.00% | 0.04% | 0.00% | 0.02% |
| GT1b - t18:0 / C24:1 | 0.00% | 0.00% | 0.00% | 0.00% | 0.00% | 0.00% | 0.00% | 0.00% | 0.00% | 0.00% | 0.00% | 0.00% | 0.00% |
| GT1b - d18:1 / C24 | 0.02% | 0.01% | 0.00% | 0.00% | 0.00% | 0.02% | 0.01% | 0.00% | 0.00% | 0.02% | 0.04% | 0.00% | 0.01% |
| GT1b - d18:1 / C23:1 | 0.00% | 0.01% | 0.00% | 0.00% | 0.02% | 0.00% | 0.01% | 0.01% | 0.00% | 0.00% | 0.02% | 0.02% | 0.02% |
| GT1b - t18:0 / C24 | 0.00% | 0.00% | 0.00% | 0.00% | 0.00% | 0.00% | 0.02% | 0.00% | 0.00% | 0.00% | 0.00% | 0.00% | 0.00% |
| GT1b - d18:1 / C23 | 0.03% | 0.05% | 0.02% | 0.00% | 0.00% | 0.05% | 0.00% | 0.03% | 0.00% | 0.02% | 0.03% | 0.02% | 0.03% |
| GT1b - d18:1 / C22 | 0.12% | 0.15% | 0.16% | 0.00% | 0.21% | 0.14% | 0.15% | 0.10% | 0.13% | 0.13% | 0.21% | 0.06% | 0.05% |
| GT1b-OAc - d18:1 / C22 | 0.01% | 0.04% | 0.03% | 0.00% | 0.04% | 0.03% | 0.03% | 0.00% | 0.03% | 0.01% | 0.02% | 0.01% | 0.01% |
| GT1-Lactone - d18:1 / C22 | 0.00% | 0.00% | 0.00% | 0.00% | 0.00% | 0.00% | 0.00% | 0.00% | 0.00% | 0.01% | 0.02% | 0.00% | 0.00% |
| GT1b - t18:0 / C22 | 0.00% | 0.00% | 0.00% | 0.00% | 0.00% | 0.00% | 0.19% | 0.00% | 0.00% | 0.02% | 0.02% | 0.00% | 0.00% |
| GT1b - d18:1 / C21 | 0.05% | 0.07% | 0.05% | 0.00% | 0.09% | 0.06% | 0.04% | 0.04% | 0.04% | 0.04% | 0.04% | 0.03% | 0.02% |
| GT1b - d18:2 / C20 | 0.00% | 0.06% | 0.07% | 0.47% | 0.00% | 0.06% | 0.08% | 0.00% | 0.02% | 0.05% | 0.09% | 0.05% | 0.06% |
| GT1b - d18:1 / C20 | 4.09% | 5.11% | 5.92% | 1.53% | 6.12% | 4.96% | 4.42% | 1.55% | 2.95% | 3.32% | 3.31% | 2.53% | 2.22% |
| GT1a - d18:1 / C20 | 0.41% | 0.32% | 0.40% | 0.16% | 0.68% | 0.54% | 0.50% | 0.15% | 0.22% | 0.42% | 0.38% | 0.20% | 0.17% |
| GT1b-OAc - d18:1 / C20 | 0.40% | 1.09% | 0.88% | 0.06% | 0.71% | 0.38% | 0.29% | 0.10% | 0.30% | 0.00% | 0.11% | 0.00% | 0.15% |
| GT1-Lactone - d18:1 / C20 | 0.03% | 0.03% | 0.04% | 0.00% | 0.02% | 0.04% | 0.04% | 0.00% | 0.00% | 0.07% | 0.12% | 0.02% | 0.01% |
| GT1b - d20:1 / C18 | 0.68% | 0.79% | 0.79% | 0.07% | 1.12% | 0.51% | 0.52% | 0.26% | 0.50% | 0.31% | 0.36% | 0.46% | 0.33% |
| GT1b - t18:0 / C20 | 0.05% | 0.06% | 0.05% | 0.00% | 0.07% | 0.07% | 0.81% | 0.00% | 0.02% | 0.04% | 0.04% | 0.00% | 0.03% |
| GT1b - d18:1 / C19 | 0.07% | 0.11% | 0.13% | 0.08% | 0.12% | 0.09% | 0.11% | 0.03% | 0.04% | 0.06% | 0.05% | 0.06% | 0.05% |
| GT1b - d18:2 / C18 | 0.00% | 0.00% | 0.00% | 0.64% | 0.00% | 0.00% | 0.00% | 0.00% | 0.00% | 0.00% | 0.00% | 0.00% | 0.00% |
| GT1b - d18:1 / C18 | 0.69% | 1.09% | 1.31% | 6.21% | 1.53% | 0.59% | 2.45% | 0.61% | 0.52% | 0.51% | 0.63% | 1.02% | 0.81% |
| GT1a - d18:1 / C18 | 0.10% | 0.12% | 0.12% | 0.42% | 0.12% | 0.09% | 0.33% | 0.05% | 0.04% | 0.00% | 0.00% | 0.14% | 0.11% |
| GT1b-OAc - d18:1 / C18 | 0.05% | 0.15% | 0.28% | 0.26% | 0.16% | 0.08% | 0.13% | 0.00% | 0.00% | 0.04% | 0.02% | 0.10% | 0.06% |
| GT1-Lactone - d18:1 / C18 | 0.00% | 0.00% | 0.00% | 0.18% | 0.00% | 0.00% | 0.03% | 0.00% | 0.00% | 0.00% | 0.00% | 0.00% | 0.00% |
| GT1b - d20:1 / C16 | 0.07% | 0.21% | 0.14% | 0.31% | 0.30% | 0.26% | 0.18% | 0.09% | 0.08% | 0.20% | 0.25% | 0.10% | 0.12% |
| GT1b - d18:2 / C17 | 0.02% | 0.00% | 0.00% | 0.00% | 0.00% | 0.02% | 0.00% | 0.00% | 0.00% | 0.01% | 0.00% | 0.00% | 0.00% |
| GT1b - t18:0 / C18 | 0.00% | 0.00% | 0.00% | 0.00% | 0.00% | 0.00% | 0.10% | 0.00% | 0.00% | 0.00% | 0.00% | 0.00% | 0.00% |
| GT1b - d18:0 / C18 | 0.12% | 0.14% | 0.13% | 0.00% | 0.26% | 0.12% | 0.21% | 0.06% | 0.06% | 0.08% | 0.08% | 0.13% | 0.13% |
| GT1b - d18:1 / C17 | 0.00% | 0.00% | 0.00% | 0.00% | 0.00% | 0.01% | 0.00% | 0.00% | 0.00% | 0.00% | 0.00% | 0.00% | 0.00% |
| GT1b - d18:1 / C16 | 0.01% | 0.01% | 0.00% | 0.00% | 0.00% | 0.00% | 0.00% | 0.00% | 0.01% | 0.01% | 0.01% | 0.00% | 0.00% |
| GT1b-OAc - d18:1 / C16 | 0.00% | 0.00% | 0.00% | 0.00% | 0.00% | 0.00% | 0.02% | 0.00% | 0.00% | 0.00% | 0.00% | 0.00% | 0.00% |
| GT1-Lactone - d18:1 / C16 | 0.00% | 0.00% | 0.00% | 0.00% | 0.00% | 0.00% | 0.00% | 0.00% | 0.00% | 0.00% | 0.00% | 0.00% | 0.00% |
| GT1b - d18:1 / C14 | 0.00% | 0.00% | 0.00% | 0.00% | 0.00% | 0.00% | 0.00% | 0.00% | 0.00% | 0.00% | 0.00% | 0.00% | 0.00% |
| GT3 - d18:2 / C20 | 0.00% | 0.00% | 0.00% | 0.00% | 0.00% | 0.01% | 0.00% | 0.00% | 0.00% | 0.00% | 0.01% | 0.00% | 0.00% |
| GT3 - d18:1 / C18 | 0.00% | 0.00% | 0.00% | 0.00% | 0.16% | 0.00% | 0.24% | 0.14% | 0.00% | 0.00% | 0.00% | 0.00% | 0.00% |
| GD1a - d18:1 / C26:1 | 0.10% | 0.06% | 0.06% | 0.00% | 0.06% | 0.17% | 0.00% | 0.00% | 0.00% | 0.09% | 0.16% | 0.10% | 0.05% |
| GD1a - t18:0 / C26:1 | 0.00% | 0.00% | 0.00% | 0.00% | 0.00% | 0.00% | 0.00% | 0.00% | 0.00% | 0.00% | 0.00% | 0.00% | 0.02% |
| GD1a - d18:1 / C26 | 0.01% | 0.00% | 0.00% | 0.00% | 0.00% | 0.02% | 0.00% | 0.00% | 0.00% | 0.00% | 0.02% | 0.00% | 0.00% |
| GD1a - d18:1 / C25:1 | 0.02% | 0.00% | 0.02% | 0.00% | 0.02% | 0.05% | 0.00% | 0.00% | 0.00% | 0.01% | 0.04% | 0.04% | 0.04% |
| GD1a - d18:1 / C25 | 0.04% | 0.00% | 0.02% | 0.00% | 0.01% | 0.08% | 0.00% | 0.00% | 0.01% | 0.04% | 0.06% | 0.04% | 0.03% |
| GD1a - d18:1 / C24:1 | 0.00% | 0.00% | 0.00% | 0.00% | 0.00% | 0.04% | 0.00% | 0.00% | 0.01% | 0.00% | 0.32% | 0.00% | 0.33% |
| GD1a - t18:0 / C24:1 | 0.00% | 0.00% | 0.11% | 0.00% | 0.00% | 0.00% | 0.00% | 0.07% | 0.00% | 0.00% | 0.00% | 0.09% | 0.00% |
| GD1a - d18:1 / C24 | 0.08% | 0.04% | 0.05% | 0.00% | 0.06% | 0.15% | 0.05% | 0.03% | 0.03% | 0.12% | 0.18% | 0.08% | 0.08% |
| GD1a - d18:1 / C23:1 | 0.01% | 0.00% | 0.00% | 0.00% | 0.00% | 0.00% | 0.00% | 0.00% | 0.00% | 0.01% | 0.00% | 0.00% | 0.00% |
| GD1a - d18:1 / C23 | 0.07% | 0.00% | 0.00% | 0.00% | 0.00% | 0.13% | 0.00% | 0.00% | 0.00% | 0.06% | 0.10% | 0.00% | 0.08% |
| GD1a - d18:1 / C22:1 | 0.00% | 0.00% | 0.00% | 0.00% | 0.00% | 0.00% | 0.00% | 0.00% | 0.01% | 0.00% | 0.00% | 0.02% | 0.00% |
| GD1a - d18:1 / C22 | 1.90% | 1.14% | 1.12% | 0.14% | 1.07% | 2.22% | 1.17% | 0.48% | 0.68% | 1.97% | 2.33% | 1.00% | 1.10% |
| GD1a-OAc - d18:1 / C22 | 0.00% | 0.06% | 0.06% | 0.00% | 0.02% | 0.06% | 0.00% | 0.00% | 0.03% | 0.00% | 0.00% | 0.05% | 0.00% |
| GD1-Lactone - d18:1 / C22 | 0.00% | 0.00% | 0.00% | 0.00% | 0.00% | 0.00% | 0.00% | 0.00% | 0.00% | 0.01% | 0.13% | 0.00% | 0.00% |
| GD1a - t18:0 / C22 | 0.00% | 0.00% | 0.00% | 0.00% | 0.00% | 0.00% | 0.16% | 0.00% | 0.00% | 0.00% | 0.00% | 0.00% | 0.00% |
| GD1a - d18:1 / C21 | 0.06% | 0.09% | 0.13% | 0.00% | 0.14% | 0.06% | 0.00% | 0.00% | 0.10% | 0.01% | 0.00% | 0.00% | 0.00% |
| GD1a - d18:2 / C20 | 1.07% | 0.62% | 0.58% | 0.59% | 0.41% | 0.00% | 0.65% | 0.00% | 0.37% | 0.86% | 0.93% | 0.60% | 0.83% |
| GD1b - d18:1 / C20 | 18.26% | 18.89% | 11.07% | 1.01% | 17.03% | 16.76% | 14.06% | 5.15% | 9.83% | 14.61% | 13.41% | 18.26% | 17.78% |
| GD1a - d18:1 / C20 | 8.50% | 0.00% | 8.90% | 0.70% | 0.00% | 8.86% | 0.00% | 0.00% | 0.00% | 7.87% | 8.52% | 0.00% | 0.00% |
| GD1b-OAc - d18:1 / C20 | 0.92% | 1.24% | 0.92% | 0.00% | 0.45% | 0.91% | 0.57% | 0.14% | 0.32% | 0.65% | 0.26% | 0.59% | 0.69% |
| GD1-Lactone - d18:1 / C20 | 0.21% | 0.15% | 0.28% | 0.00% | 0.12% | 0.37% | 0.23% | 0.06% | 0.10% | 0.88% | 1.13% | 0.15% | 0.09% |
| GD1b - d20:1 / C18 | 2.12% | 2.41% | 2.92% | 0.15% | 2.68% | 2.38% | 1.15% | 0.83% | 1.36% | 2.63% | 2.68% | 2.43% | 1.36% |
| GD1b - t18:0 / C20 | 0.00% | 0.15% | 0.14% | 0.00% | 0.10% | 0.26% | 2.08% | 0.05% | 0.05% | 0.20% | 0.21% | 0.13% | 0.12% |
| GD1b - d18:1 / C19 | 0.64% | 0.00% | 0.64% | 0.00% | 0.00% | 0.70% | 0.00% | 0.00% | 0.00% | 0.00% | 0.38% | 0.36% | 0.00% |
| GD1b - d18:2 / C18 | 0.22% | 0.19% | 0.24% | 6.04% | 0.18% | 0.22% | 0.29% | 0.13% | 0.16% | 0.21% | 0.25% | 0.21% | 0.27% |
| GD1b - d18:1 / C18 | 16.73% | 12.87% | 13.17% | 2.50% | 9.04% | 16.50% | 24.46% | 5.01% | 5.57% | 14.84% | 17.09% | 16.28% | 21.46% |
| GD1-Lactone - d18:1 / C18 | 0.11% | 0.09% | 0.13% | 0.10% | 0.04% | 0.18% | 0.28% | 0.00% | 0.04% | 0.45% | 0.79% | 0.09% | 0.11% |
| GD1b - d20:1 / C16 | 2.23% | 1.28% | 1.73% | 0.36% | 1.29% | 2.66% | 3.28% | 0.75% | 0.70% | 2.20% | 2.39% | 2.05% | 2.59% |
| GD1b - t18:0 / C18 | 0.14% | 0.00% | 0.00% | 0.00% | 0.06% | 0.22% | 2.42% | 0.00% | 0.04% | 0.16% | 0.17% | 0.00% | 0.18% |
| GD1b - d18:0 / C18 | 2.03% | 1.33% | 1.02% | 0.32% | 1.04% | 1.96% | 1.51% | 0.52% | 0.56% | 1.81% | 1.58% | 1.55% | 1.95% |
| GD1b - d18:1 / C17 | 0.00% | 0.00% | 0.06% | 0.00% | 0.03% | 0.04% | 0.07% | 0.03% | 0.00% | 0.03% | 0.04% | 0.04% | 0.04% |
| GD1b - d18:1 / C16 | 0.00% | 0.03% | 0.04% | 0.65% | 0.00% | 0.00% | 0.08% | 0.00% | 0.00% | 0.00% | 0.00% | 0.00% | 0.00% |
| GD1b-OAc - d18:1 / C16 | 0.02% | 0.00% | 0.11% | 0.15% | 0.11% | 0.31% | 0.00% | 0.00% | 0.07% | 0.20% | 0.20% | 0.00% | 0.00% |
| GD1b - d18:0 / C14 | 0.00% | 0.00% | 0.00% | 0.00% | 0.00% | 0.00% | 0.00% | 0.00% | 0.00% | 0.02% | 0.03% | 0.00% | 0.00% |
| GD2 - d18:1 / C24:1 | 0.02% | 0.00% | 0.00% | 0.00% | 0.00% | 0.00% | 0.00% | 0.02% | 0.00% | 0.00% | 0.00% | 0.00% | 0.00% |
| GD2 - d18:1 / C22 | 0.00% | 0.00% | 0.00% | 0.00% | 0.00% | 0.00% | 0.00% | 0.00% | 0.00% | 0.00% | 0.16% | 0.00% | 0.00% |
| GD2 - d18:1 / C20 | 1.05% | 0.93% | 0.93% | 0.00% | 0.66% | 0.66% | 0.75% | 0.00% | 0.38% | 0.92% | 1.20% | 1.81% | 1.03% |
| GD2 - d18:1 / C19 | 0.03% | 0.02% | 0.02% | 0.00% | 0.01% | 0.02% | 0.02% | 0.00% | 0.01% | 0.03% | 0.03% | 0.04% | 0.02% |
| GD2 - d18:1 / C18 | 1.08% | 0.85% | 0.72% | 0.10% | 0.65% | 0.70% | 1.86% | 0.22% | 0.45% | 0.89% | 1.22% | 2.16% | 1.53% |
| GD2 - d18:0 / C18 | 0.12% | 0.07% | 0.05% | 0.00% | 0.06% | 0.06% | 0.12% | 0.00% | 0.02% | 0.09% | 0.10% | 0.21% | 0.12% |
| GD3 - d18:1 / C24:1 | 0.00% | 0.00% | 0.00% | 0.00% | 0.03% | 0.00% | 0.10% | 0.16% | 0.00% | 0.00% | 0.00% | 0.00% | 0.00% |
| GD3 - d18:1 / C22 | 0.00% | 0.00% | 0.00% | 0.00% | 0.00% | 0.00% | 0.00% | 0.00% | 0.00% | 0.00% | 0.11% | 0.00% | 0.00% |
| GD3 - d18:2 / C20 | 0.00% | 0.00% | 0.00% | 0.00% | 0.00% | 0.00% | 0.00% | 0.04% | 0.00% | 0.00% | 0.00% | 0.00% | 0.00% |
| GD3 - d18:1 / C20 | 0.54% | 0.53% | 0.54% | 0.00% | 0.50% | 0.30% | 0.78% | 0.37% | 0.23% | 0.44% | 0.00% | 0.86% | 0.48% |
| GD3 - d18:1 / C19 | 0.02% | 0.02% | 0.02% | 0.00% | 0.04% | 0.02% | 0.04% | 0.05% | 0.02% | 0.03% | 0.02% | 0.03% | 0.02% |
| GD3 - d18:2 / C18 | 0.00% | 0.00% | 0.00% | 0.17% | 0.13% | 0.06% | 0.12% | 0.00% | 0.00% | 0.00% | 0.00% | 0.11% | 0.08% |
| GD3 - d18:1 / C18 | 1.27% | 1.78% | 1.15% | 0.17% | 0.00% | 1.79% | 0.45% | 0.00% | 1.45% | 1.04% | 1.40% | 0.00% | 0.00% |
| GD3 - t18:0 / C18 | 0.00% | 0.00% | 0.00% | 0.00% | 0.00% | 0.01% | 0.20% | 0.00% | 0.00% | 0.00% | 0.00% | 0.02% | 0.00% |
| GD3 - d18:0 / C18 | 0.02% | 0.00% | 0.09% | 0.00% | 0.10% | 0.06% | 0.09% | 0.23% | 0.00% | 0.00% | 0.00% | 0.14% | 0.05% |
| GD3 - d18:1 / C17 | 0.02% | 0.03% | 0.01% | 0.00% | 0.04% | 0.01% | 0.03% | 0.06% | 0.00% | 0.00% | 0.00% | 0.00% | 0.00% |
| GD3 - d18:1 / C16 | 0.01% | 0.02% | 0.00% | 0.00% | 0.04% | 0.02% | 0.04% | 0.08% | 0.02% | 0.00% | 0.01% | 0.03% | 0.01% |
| GD3 - d18:0 / C16 | 0.00% | 0.00% | 0.00% | 0.00% | 0.00% | 0.00% | 0.00% | 0.02% | 0.00% | 0.00% | 0.00% | 0.00% | 0.00% |
| GM1a - d18:1 / C26:1 | 0.05% | 0.02% | 0.03% | 0.00% | 0.00% | 0.09% | 0.00% | 0.00% | 0.02% | 0.06% | 0.07% | 0.05% | 0.06% |
| GM1a - d18:1 / C26 | 0.00% | 0.00% | 0.00% | 0.00% | 0.00% | 0.01% | 0.00% | 0.00% | 0.00% | 0.01% | 0.00% | 0.00% | 0.00% |
| GM1a - d18:1 / C25:1 | 0.00% | 0.00% | 0.00% | 0.00% | 0.00% | 0.06% | 0.00% | 0.00% | 0.00% | 0.00% | 0.05% | 0.03% | 0.03% |
| GM1a - d18:1 / C25 | 0.00% | 0.00% | 0.00% | 0.00% | 0.00% | 0.05% | 0.00% | 0.00% | 0.00% | 0.03% | 0.03% | 0.03% | 0.02% |
| GM1a - d18:1 / C24 OH | 0.00% | 0.00% | 0.00% | 0.00% | 0.00% | 0.06% | 0.00% | 0.00% | 0.00% | 0.04% | 0.06% | 0.00% | 0.00% |
| GM1a - d18:1 / C23 | 0.00% | 0.00% | 0.00% | 0.00% | 0.00% | 0.12% | 0.00% | 0.00% | 0.00% | 0.00% | 0.06% | 0.00% | 0.00% |
| GM1a - d18:1 / C22 | 0.47% | 0.16% | 0.22% | 0.12% | 0.00% | 0.61% | 0.14% | 0.00% | 0.12% | 0.81% | 0.76% | 0.35% | 0.36% |
| GM1a - d18:1 / C21 | 0.09% | 0.00% | 0.07% | 0.00% | 0.04% | 0.15% | 0.00% | 0.00% | 0.00% | 0.15% | 0.12% | 0.06% | 0.06% |
| GM1a - d18:2 / C20 | 0.00% | 0.37% | 0.00% | 0.23% | 0.23% | 0.00% | 0.25% | 0.10% | 0.00% | 0.87% | 0.65% | 0.64% | 0.00% |
| GM1a - d18:1 / C20 | 5.34% | 5.05% | 5.19% | 13.47% | 2.49% | 0.27% | 0.26% | 2.72% | 4.73% | 6.95% | 6.05% | 6.45% | 5.10% |
| GM1a - d20:1 / C18 | 1.26% | 0.73% | 0.87% | 0.13% | 0.00% | 0.00% | 0.00% | 0.19% | 0.67% | 1.38% | 0.89% | 1.00% | 0.00% |
| GM1a - t18:0 / C20 | 0.03% | 0.00% | 0.00% | 0.00% | 0.04% | 0.13% | 0.52% | 0.00% | 0.03% | 0.09% | 0.00% | 0.00% | 0.00% |
| GM1a - d18:1 / C19 | 0.29% | 0.24% | 0.19% | 0.00% | 0.24% | 0.39% | 0.15% | 0.22% | 0.17% | 0.35% | 0.26% | 0.27% | 0.24% |
| GM1a - d18:2 / C18 | 1.36% | 1.02% | 0.68% | 2.52% | 0.46% | 1.51% | 0.67% | 0.47% | 0.55% | 1.60% | 1.26% | 1.33% | 1.87% |
| GM1a - d18:1 / C18 | 12.37% | 8.47% | 18.13% | 51.35% | 12.75% | 14.46% | 9.66% | 24.74% | 19.14% | 16.71% | 13.23% | 18.13% | 17.46% |
| GM1a - d20:1 / C16 | 0.72% | 0.59% | 0.67% | 0.27% | 0.53% | 0.79% | 0.60% | 1.21% | 0.66% | 1.58% | 1.11% | 1.54% | 2.24% |
| GM1a - t18:0 / C18 | 0.11% | 0.05% | 0.00% | 0.00% | 0.00% | 0.22% | 0.88% | 0.10% | 0.00% | 0.13% | 0.06% | 0.07% | 0.16% |
| GM1a - d18:0 / C18 | 1.05% | 0.63% | 0.37% | 0.12% | 0.48% | 1.09% | 0.54% | 1.25% | 0.51% | 1.49% | 0.91% | 1.24% | 1.53% |
| GM1a - d18:1 / C17 | 0.09% | 0.11% | 0.06% | 0.00% | 0.07% | 0.16% | 0.05% | 0.14% | 0.05% | 0.07% | 0.07% | 0.07% | 0.10% |
| GM1a - d18:1 / C16 | 0.46% | 0.59% | 0.39% | 0.30% | 0.33% | 0.73% | 0.42% | 0.56% | 0.29% | 0.43% | 0.37% | 0.46% | 0.42% |
| GM1a - d18:0 / C16 | 0.00% | 0.00% | 0.00% | 0.00% | 0.01% | 0.03% | 0.01% | 0.03% | 0.00% | 0.02% | 0.01% | 0.00% | 0.03% |
| GM1a - d18:0 / C14 | 0.00% | 0.00% | 0.00% | 0.00% | 0.00% | 0.02% | 0.00% | 0.05% | 0.00% | 0.00% | 0.00% | 0.00% | 0.02% |
| GM2 - d18:1 / C21 | 0.00% | 0.00% | 0.00% | 0.00% | 0.00% | 0.00% | 0.00% | 0.00% | 0.00% | 0.01% | 0.00% | 0.00% | 0.00% |
| GM2 - d18:2 / C20 | 0.03% | 0.00% | 0.00% | 0.00% | 0.00% | 0.00% | 0.00% | 0.00% | 0.00% | 0.00% | 0.00% | 0.02% | 0.02% |
| GM2 - d18:1 / C20 | 0.19% | 0.11% | 0.12% | 0.04% | 0.00% | 0.30% | 0.00% | 0.00% | 0.11% | 0.00% | 0.00% | 0.42% | 0.26% |
| GM2 - t18:0 / C20 | 0.01% | 0.00% | 0.00% | 0.00% | 0.00% | 0.01% | 0.04% | 0.00% | 0.00% | 0.01% | 0.00% | 0.00% | 0.00% |
| GM2 - d18:1 / C19 | 0.02% | 0.01% | 0.01% | 0.00% | 0.01% | 0.02% | 0.01% | 0.00% | 0.00% | 0.02% | 0.02% | 0.03% | 0.02% |
| GM2 - d18:2 / C18 | 0.06% | 0.03% | 0.03% | 0.05% | 0.04% | 0.09% | 0.00% | 0.03% | 0.04% | 0.05% | 0.07% | 0.08% | 0.09% |
| GM2 - d18:1 / C18 | 0.77% | 0.58% | 0.44% | 0.09% | 0.50% | 0.95% | 0.75% | 0.44% | 0.40% | 0.95% | 1.08% | 1.90% | 1.67% |
| GM2 - t18:0 / C18 | 0.01% | 0.00% | 0.00% | 0.00% | 0.00% | 0.04% | 0.07% | 0.00% | 0.00% | 0.02% | 0.02% | 0.02% | 0.00% |
| GM2 - d18:0 / C18 | 0.00% | 0.02% | 0.01% | 0.00% | 0.03% | 0.00% | 0.03% | 0.04% | 0.02% | 0.05% | 0.04% | 0.13% | 0.09% |
| GM2 - d18:1 / C17 | 0.01% | 0.00% | 0.00% | 0.00% | 0.00% | 0.02% | 0.00% | 0.00% | 0.00% | 0.00% | 0.00% | 0.00% | 0.00% |
| GM2 - d18:1 / C16 | 0.05% | 0.06% | 0.04% | 0.00% | 0.04% | 0.10% | 0.05% | 0.07% | 0.05% | 0.03% | 0.04% | 0.05% | 0.00% |
| GM2 - d18:0 / C14 | 0.00% | 0.00% | 0.00% | 0.00% | 0.00% | 0.00% | 0.00% | 0.00% | 0.00% | 0.00% | 0.00% | 0.00% | 0.00% |
| GM3 - d18:1 / C26:1 | 0.00% | 0.00% | 0.01% | 0.00% | 0.00% | 0.00% | 0.00% | 0.00% | 0.00% | 0.00% | 0.00% | 0.00% | 0.00% |
| GM3 - d18:1 / C26 | 0.00% | 0.00% | 0.00% | 0.00% | 0.00% | 0.00% | 0.00% | 0.00% | 0.00% | 0.00% | 0.00% | 0.02% | 0.00% |
| GM3 - d18:1 / C25:1 | 0.00% | 0.01% | 0.04% | 0.00% | 0.02% | 0.00% | 0.00% | 0.01% | 0.03% | 0.00% | 0.00% | 0.00% | 0.00% |
| GM3 - d18:1 / C24:1 | 0.00% | 0.00% | 0.11% | 0.00% | 0.09% | 0.02% | 0.06% | 0.11% | 0.17% | 0.00% | 0.06% | 0.16% | 0.08% |
| GM3 - d18:1 / C24 OH | 0.00% | 0.00% | 0.00% | 0.00% | 0.00% | 0.00% | 0.00% | 0.00% | 0.00% | 0.00% | 0.01% | 0.03% | 0.02% |
| GM3 - d18:1 / C24 | 0.02% | 0.00% | 0.02% | 0.00% | 0.00% | 0.01% | 0.00% | 0.00% | 0.02% | 0.01% | 0.06% | 0.18% | 0.11% |
| GM3 - d18:1 / C23 | 0.00% | 0.00% | 0.00% | 0.00% | 0.00% | 0.00% | 0.01% | 0.00% | 0.00% | 0.00% | 0.01% | 0.03% | 0.02% |
| GM3 - d18:1 / C22:1 | 0.00% | 0.00% | 0.00% | 0.00% | 0.00% | 0.00% | 0.00% | 0.00% | 0.00% | 0.00% | 0.00% | 0.02% | 0.02% |
| GM3 - d18:1 / C22 | 0.00% | 0.00% | 0.00% | 0.00% | 0.00% | 0.02% | 0.01% | 0.00% | 0.01% | 0.02% | 0.06% | 0.15% | 0.10% |
| GM3 - d18:2 / C20 | 0.00% | 0.00% | 0.00% | 0.00% | 0.01% | 0.00% | 0.00% | 0.01% | 0.00% | 0.00% | 0.03% | 0.02% | 0.00% |
| GM3 - d18:1 / C20 | 0.22% | 0.09% | 0.10% | 0.00% | 0.03% | 0.22% | 0.18% | 0.09% | 0.08% | 0.01% | 0.03% | 0.06% | 0.22% |
| GM3 - d18:1 / C19 | 0.01% | 0.00% | 0.00% | 0.00% | 0.01% | 0.00% | 0.01% | 0.02% | 0.00% | 0.01% | 0.00% | 0.01% | 0.01% |
| GM3 - d18:2 / C18 | 0.00% | 0.02% | 0.00% | 0.00% | 0.06% | 0.00% | 0.03% | 0.09% | 0.04% | 0.01% | 0.01% | 0.00% | 0.02% |
| GM3 - d18:1 / C18 | 0.40% | 0.38% | 0.28% | 0.00% | 0.66% | 0.57% | 0.87% | 0.94% | 0.39% | 0.48% | 0.73% | 0.83% | 0.83% |
| GM3 - d18:0 / C18 | 0.00% | 0.00% | 0.00% | 0.00% | 0.00% | 0.00% | 0.00% | 0.06% | 0.01% | 0.00% | 0.00% | 0.05% | 0.04% |
| GM3 - d18:1 / C16 | 0.02% | 0.05% | 0.02% | 0.00% | 0.07% | 0.04% | 0.09% | 0.21% | 0.08% | 0.02% | 0.04% | 0.15% | 0.08% |
| Gal-Fuc-GD1a - d18:1 / C22:1 | 0.00% | 0.00% | 0.00% | 0.00% | 0.00% | 0.00% | 0.00% | 0.00% | 0.00% | 0.00% | 0.01% | 0.00% | 0.00% |
| Gal-Fuc-GD1a - d18:1 / C22 | 0.00% | 0.00% | 0.00% | 0.00% | 0.00% | 0.01% | 0.00% | 0.00% | 0.00% | 0.00% | 0.00% | 0.00% | 0.00% |
| Gal-Fuc-GD1a - d18:1 / C20 | 0.14% | 0.15% | 0.21% | 0.00% | 0.19% | 0.15% | 0.14% | 0.04% | 0.08% | 0.11% | 0.00% | 0.06% | 0.07% |
| Gal-Fuc-GD1a - d18:1 / C19 | 0.00% | 0.00% | 0.00% | 0.00% | 0.00% | 0.00% | 0.00% | 0.00% | 0.00% | 0.00% | 0.01% | 0.00% | 0.00% |
| Gal-Fuc-GD1b - d18:1 / C18 | 0.00% | 0.04% | 0.06% | 0.00% | 0.05% | 0.00% | 0.00% | 0.00% | 0.00% | 0.00% | 0.00% | 0.04% | 0.00% |
| Gal-Fuc-GD1b - d18:0 / C18 | 0.00% | 0.00% | 0.01% | 0.00% | 0.00% | 0.00% | 0.00% | 0.00% | 0.00% | 0.00% | 0.00% | 0.00% | 0.00% |
| Fuc-GD1a - d18:1 / C20 | 0.03% | 0.03% | 0.03% | 0.00% | 0.00% | 0.03% | 0.13% | 0.00% | 0.01% | 0.03% | 0.04% | 0.00% | 0.00% |
| Fuc-GD1b - d18:1 / C18 | 0.02% | 0.02% | 0.02% | 0.00% | 0.00% | 0.00% | 0.18% | 0.00% | 0.00% | 0.01% | 0.04% | 0.03% | 0.06% |
| Gal-Fuc-GM1a - d18:2 / C20 | 0.00% | 0.00% | 0.00% | 0.58% | 0.00% | 0.09% | 0.00% | 0.00% | 0.00% | 0.00% | 0.00% | 0.00% | 0.00% |
| Gal-Fuc-GM1a - d18:1 / C20 | 0.00% | 0.00% | 0.00% | 1.10% | 0.00% | 0.00% | 0.00% | 0.44% | 1.26% | 0.00% | 0.00% | 0.00% | 0.00% |
| Gal-Fuc-GM1a - d18:1 / C19 | 0.00% | 0.05% | 0.00% | 0.00% | 0.04% | 0.07% | 0.04% | 0.00% | 0.06% | 0.04% | 0.04% | 0.04% | 0.03% |
| Gal-Fuc-GM1a - d18:2 / C18 | 0.04% | 0.00% | 0.00% | 0.00% | 0.00% | 0.02% | 0.03% | 0.00% | 0.00% | 0.02% | 0.03% | 0.00% | 0.00% |
| Gal-Fuc-GM1a - d18:1 / C18 | 1.59% | 1.35% | 1.26% | 0.00% | 0.85% | 3.85% | 0.00% | 0.56% | 0.65% | 1.59% | 1.83% | 1.67% | 2.13% |
| Gal-Fuc-GM1a - d18:0 / C18 | 0.09% | 0.00% | 0.05% | 0.00% | 0.05% | 0.11% | 0.07% | 0.00% | 0.04% | 0.08% | 0.08% | 0.00% | 0.33% |
| Gal-Fuc-GM1a - d18:1 / C16 | 0.00% | 0.00% | 0.00% | 0.49% | 0.00% | 0.00% | 0.00% | 0.00% | 0.00% | 0.00% | 0.00% | 0.00% | 0.00% |
| GalNAc-Fuc-GM1a - d18:1 / C18 | 0.00% | 0.05% | 0.00% | 0.00% | 0.00% | 0.00% | 0.00% | 0.00% | 0.00% | 0.00% | 0.00% | 0.03% | 0.00% |
| GalNAc-Fuc-GM1a - t18:0 / C18 | 0.00% | 0.04% | 0.00% | 0.00% | 0.00% | 0.00% | 0.00% | 0.00% | 0.00% | 0.00% | 0.00% | 0.00% | 0.05% |
| Fuc-GM1a - d18:1 / C18 | 0.03% | 0.02% | 0.00% | 0.00% | 0.00% | 0.00% | 0.15% | 0.01% | 0.01% | 0.04% | 0.07% | 0.05% | 0.11% |
| Gal-Fuc-GA1 - d18:2 / C20 | 0.00% | 0.00% | 0.00% | 0.00% | 0.00% | 0.00% | 0.00% | 0.00% | 0.06% | 0.00% | 0.00% | 0.00% | 0.00% |
| Gal-Fuc-GA1 - d18:1 / C20 | 0.00% | 0.00% | 0.00% | 0.00% | 1.30% | 1.76% | 0.00% | 0.00% | 0.00% | 0.00% | 1.25% | 0.00% | 0.72% |
| Fuc-GA1 - d18:1 / C18 | 0.00% | 0.00% | 0.01% | 0.00% | 0.04% | 0.01% | 0.07% | 0.15% | 0.02% | 0.00% | 0.06% | 0.06% | 0.05% |
| Fuc-GA1 - d18:1 / C16 | 0.00% | 0.00% | 0.00% | 0.00% | 0.00% | 0.00% | 0.01% | 0.04% | 0.00% | 0.00% | 0.00% | 0.01% | 0.00% |
| GA1 - d18:1 / C24:1 | 0.00% | 0.00% | 0.00% | 0.00% | 0.00% | 0.00% | 0.00% | 0.00% | 0.00% | 0.00% | 0.03% | 0.06% | 0.00% |
| GA1 - d18:1 / C24 | 0.00% | 0.00% | 0.00% | 0.00% | 0.00% | 0.00% | 0.00% | 0.00% | 0.00% | 0.00% | 0.00% | 0.03% | 0.00% |
| GA1 - d18:1 / C20 | 0.14% | 0.00% | 0.12% | 0.00% | 0.00% | 0.20% | 0.03% | 0.00% | 0.00% | 0.27% | 0.24% | 0.10% | 0.09% |
| GA1 - d18:1 / C18 | 0.00% | 0.00% | 0.03% | 0.00% | 0.00% | 0.07% | 0.11% | 0.12% | 0.00% | 0.01% | 0.24% | 0.15% | 0.00% |
| GA1 - d18:1 / C16 | 0.00% | 0.00% | 0.00% | 0.00% | 0.00% | 0.04% | 0.02% | 0.00% | 0.00% | 0.00% | 0.00% | 0.21% | 0.09% |
| Lac - d18:1 / C26:1 | 0.01% | 0.01% | 0.01% | 0.00% | 0.00% | 0.00% | 0.00% | 0.01% | 0.01% | 0.01% | 0.01% | 0.00% | 0.00% |
| Lac - d18:1 / C25:1 | 0.00% | 0.02% | 0.01% | 0.00% | 0.01% | 0.00% | 0.00% | 0.00% | 0.02% | 0.01% | 0.01% | 0.00% | 0.00% |
| Lac - d18:1 / C24:1 | 0.04% | 0.07% | 0.04% | 0.04% | 0.06% | 0.01% | 0.04% | 0.06% | 0.09% | 0.03% | 0.06% | 0.06% | 0.03% |
| Lac - d18:1 / C24 | 0.00% | 0.01% | 0.00% | 0.00% | 0.00% | 0.00% | 0.00% | 0.01% | 0.01% | 0.00% | 0.00% | 0.01% | 0.00% |
| Lac - d18:1 / C22 | 0.00% | 0.00% | 0.00% | 0.00% | 0.00% | 0.00% | 0.00% | 0.00% | 0.00% | 0.00% | 0.02% | 0.02% | 0.00% |
| Lac - d18:2 / C20 | 0.00% | 0.00% | 0.01% | 0.00% | 0.00% | 0.00% | 0.00% | 0.00% | 0.00% | 0.01% | 0.01% | 0.00% | 0.00% |
| Lac - d18:1 / C20 | 0.03% | 0.00% | 0.00% | 0.00% | 0.00% | 0.00% | 0.00% | 0.00% | 0.00% | 0.06% | 0.10% | 0.05% | 0.04% |
| Lac - d18:2 / C18 | 0.01% | 0.02% | 0.01% | 0.00% | 0.00% | 0.01% | 0.02% | 0.03% | 0.00% | 0.02% | 0.02% | 0.02% | 0.03% |
| Lac - d18:1 / C18 | 0.13% | 0.16% | 0.10% | 0.06% | 0.19% | 0.12% | 0.23% | 0.39% | 0.17% | 0.22% | 0.31% | 0.21% | 0.24% |
| Lac - d18:1 / C16 | 0.03% | 0.05% | 0.02% | 0.00% | 0.06% | 0.03% | 0.06% | 0.17% | 0.05% | 0.03% | 0.04% | 0.38% | 0.12% |
| Gal - d18:1 / C26:1 | 0.06% | 0.12% | 0.00% | 0.09% | 0.07% | 0.00% | 0.05% | 0.15% | 0.13% | 0.00% | 0.00% | 0.03% | 0.02% |
| Gal - d18:1 / C26 OH | 0.03% | 0.04% | 0.02% | 0.03% | 0.02% | 0.00% | 0.02% | 0.03% | 0.02% | 0.02% | 0.01% | 0.00% | 0.00% |
| Gal - d18:1 / C25:1 | 0.10% | 0.29% | 0.16% | 0.14% | 0.15% | 0.02% | 0.09% | 0.23% | 0.25% | 0.05% | 0.04% | 0.04% | 0.03% |
| Gal - d18:1 / C25 OH | 0.10% | 0.27% | 0.14% | 0.17% | 0.13% | 0.03% | 0.11% | 0.17% | 0.19% | 0.09% | 0.05% | 0.05% | 0.02% |
| Gal - d18:1 / C25 | 0.01% | 0.04% | 0.01% | 0.00% | 0.01% | 0.00% | 0.01% | 0.06% | 0.03% | 0.01% | 0.00% | 0.00% | 0.00% |
| Gal - d18:1 / C24:1 | 0.44% | 1.56% | 0.67% | 0.56% | 0.95% | 0.00% | 0.54% | 1.88% | 1.29% | 0.20% | 0.16% | 0.20% | 0.12% |
| Gal - d18:1 / C24 OH | 0.44% | 1.38% | 0.78% | 0.98% | 0.81% | 0.12% | 0.57% | 1.32% | 1.07% | 0.35% | 0.21% | 0.22% | 0.12% |
| Gal - d18:1 / C24 | 0.02% | 0.10% | 0.03% | 0.07% | 0.05% | 0.00% | 0.03% | 0.13% | 0.11% | 0.00% | 0.00% | 0.00% | 0.00% |
| Gal - t18:0 / C24 | 0.02% | 0.05% | 0.00% | 0.03% | 0.02% | 0.00% | 0.00% | 0.05% | 0.04% | 0.01% | 0.00% | 0.00% | 0.00% |
| Gal - d18:1 / C23 | 0.04% | 0.11% | 0.06% | 0.06% | 0.05% | 0.00% | 0.04% | 0.11% | 0.09% | 0.02% | 0.02% | 0.01% | 0.00% |
| Gal - d18:1 / C22 | 0.00% | 0.08% | 0.00% | 0.00% | 0.05% | 0.00% | 0.00% | 0.16% | 0.09% | 0.00% | 0.00% | 0.00% | 0.00% |
| Gal - d18:1 / C20 | 0.00% | 0.00% | 0.00% | 0.00% | 0.07% | 0.00% | 0.00% | 0.18% | 0.09% | 0.00% | 0.00% | 0.00% | 0.00% |
| Gal - d18:1 / C18 | 0.36% | 1.67% | 0.55% | 0.20% | 2.17% | 0.04% | 0.78% | 4.89% | 2.05% | 0.09% | 0.23% | 0.12% | 0.07% |
| Gal - d18:1 / C17 | 0.00% | 0.01% | 0.00% | 0.00% | 0.02% | 0.00% | 0.00% | 0.07% | 0.02% | 0.00% | 0.00% | 0.00% | 0.00% |
| Gal - d18:1 / C16 | 0.01% | 0.04% | 0.02% | 0.00% | 0.06% | 0.00% | 0.01% | 0.13% | 0.05% | 0.01% | 0.00% | 0.01% | 0.00% |
| SM3 - d18:1 / C26:1 | 0.00% | 0.02% | 0.01% | 0.00% | 0.01% | 0.00% | 0.01% | 0.00% | 0.00% | 0.00% | 0.00% | 0.00% | 0.00% |
| SM3 - d18:1 / C25:1 | 0.00% | 0.02% | 0.01% | 0.00% | 0.02% | 0.00% | 0.01% | 0.01% | 0.02% | 0.00% | 0.00% | 0.00% | 0.00% |
| SM3 - d18:1 / C25 | 0.00% | 0.00% | 0.00% | 0.00% | 0.00% | 0.00% | 0.00% | 0.00% | 0.00% | 0.00% | 0.00% | 0.00% | 0.00% |
| SM3 - d18:1 / C24:1 | 0.00% | 0.04% | 0.04% | 0.00% | 0.08% | 0.00% | 0.01% | 0.06% | 0.07% | 0.00% | 0.00% | 0.00% | 0.00% |
| SM3 - d18:1 / C24 | 0.00% | 0.01% | 0.00% | 0.00% | 0.03% | 0.00% | 0.00% | 0.00% | 0.02% | 0.00% | 0.00% | 0.00% | 0.00% |
| SM3 - d18:1 / C23 | 0.00% | 0.00% | 0.00% | 0.00% | 0.01% | 0.00% | 0.00% | 0.01% | 0.00% | 0.00% | 0.00% | 0.00% | 0.00% |
| SM3 - d18:1 / C22 | 0.00% | 0.00% | 0.00% | 0.00% | 0.00% | 0.00% | 0.00% | 0.00% | 0.00% | 0.00% | 0.00% | 0.00% | 0.00% |
| SM3 - d18:1 / C20 | 0.02% | 0.01% | 0.02% | 0.00% | 0.00% | 0.02% | 0.00% | 0.00% | 0.01% | 0.00% | 0.00% | 0.01% | 0.00% |
| SM3 - d18:1 / C18 | 0.04% | 0.06% | 0.04% | 0.00% | 0.09% | 0.06% | 0.07% | 0.10% | 0.07% | 0.05% | 0.04% | 0.03% | 0.03% |
| SM4 - d18:1 / C26:1 | 0.27% | 1.19% | 0.62% | 0.22% | 2.49% | 0.22% | 0.80% | 2.72% | 2.37% | 0.21% | 0.21% | 0.46% | 0.51% |
| SM4 - d18:1 / C26 OH | 0.06% | 0.15% | 0.10% | 0.05% | 0.24% | 0.07% | 0.12% | 0.22% | 0.21% | 0.06% | 0.04% | 0.06% | 0.07% |
| SM4 - d18:1 / C26 | 0.02% | 0.07% | 0.02% | 0.00% | 0.17% | 0.02% | 0.07% | 0.27% | 0.17% | 0.02% | 0.01% | 0.02% | 0.02% |
| SM4 - d18:1 / C25:1 | 0.43% | 1.68% | 1.23% | 0.00% | 3.21% | 0.39% | 1.25% | 3.02% | 3.33% | 0.30% | 0.34% | 0.68% | 0.63% |
| SM4 - d18:1 / C25 OH | 0.22% | 0.70% | 0.52% | 0.21% | 1.37% | 0.25% | 0.54% | 0.96% | 1.15% | 0.24% | 0.16% | 0.27% | 0.25% |
| SM4 - d18:1 / C25 | 0.09% | 0.42% | 0.15% | 0.10% | 0.89% | 0.07% | 0.25% | 1.28% | 1.33% | 0.10% | 0.06% | 0.13% | 0.13% |
| SM4 - d18:1 / C24:1 | 0.94% | 6.34% | 3.28% | 0.79% | 0.00% | 0.00% | 3.65% | 0.00% | 12.37% | 0.69% | 0.74% | 1.59% | 1.47% |
| SM4 - t18:0 / C25 | 0.00% | 0.02% | 0.00% | 0.00% | 0.05% | 0.00% | 0.01% | 0.04% | 0.05% | 0.01% | 0.00% | 0.00% | 0.01% |
| SM4 - d18:1 / C24 OH | 0.74% | 2.52% | 1.72% | 0.89% | 5.16% | 0.78% | 2.21% | 5.74% | 5.05% | 0.84% | 0.62% | 1.00% | 0.88% |
| SM4 - d18:1 / C24 | 0.25% | 1.26% | 0.55% | 0.29% | 2.56% | 0.20% | 0.62% | 4.44% | 3.42% | 0.24% | 0.15% | 0.30% | 0.35% |
| SM4 - d18:1 / C23:1 | 0.03% | 0.16% | 0.10% | 0.00% | 0.00% | 0.00% | 0.09% | 0.10% | 0.06% | 0.02% | 0.02% | 0.05% | 0.04% |
| SM4 - t18:0 / C24 | 0.03% | 0.14% | 0.10% | 0.00% | 0.27% | 0.04% | 0.06% | 0.38% | 0.31% | 0.04% | 0.04% | 0.05% | 0.04% |
| SM4 - d18:1 / C23 | 0.06% | 0.41% | 0.21% | 0.11% | 0.56% | 0.05% | 0.19% | 1.15% | 0.80% | 0.08% | 0.05% | 0.08% | 0.10% |
| SM4 - d18:1 / C22:1 | 0.00% | 0.16% | 0.00% | 0.00% | 0.39% | 0.00% | 0.00% | 0.52% | 0.33% | 0.00% | 0.00% | 0.00% | 0.00% |
| SM4 - d18:1 / C22 | 0.00% | 0.19% | 0.11% | 0.00% | 0.36% | 0.00% | 0.18% | 0.90% | 0.52% | 0.04% | 0.00% | 0.00% | 0.00% |
| SM4 - d18:1 / C21 | 0.00% | 0.05% | 0.02% | 0.00% | 0.00% | 0.00% | 0.00% | 0.10% | 0.06% | 0.00% | 0.00% | 0.00% | 0.00% |
| SM4 - d18:1 / C19 | 0.00% | 0.04% | 0.00% | 0.00% | 0.08% | 0.00% | 0.00% | 0.16% | 0.07% | 0.00% | 0.00% | 0.00% | 0.00% |
| SM4 - d18:2 / C18 | 0.07% | 0.28% | 0.08% | 0.04% | 0.64% | 0.08% | 0.17% | 1.15% | 0.31% | 0.04% | 0.04% | 0.13% | 0.10% |
| SM4 - d18:1 / C18 OH | 0.06% | 0.19% | 0.09% | 0.00% | 0.60% | 0.08% | 0.17% | 0.89% | 0.28% | 0.05% | 0.07% | 0.05% | 0.04% |
| SM4 - d18:1 / C18 | 0.31% | 1.73% | 0.50% | 0.08% | 4.93% | 0.00% | 0.97% | 11.89% | 3.29% | 0.16% | 0.14% | 0.36% | 0.27% |
| SM4 - d18:1 / C17 | 0.00% | 0.02% | 0.01% | 0.00% | 0.05% | 0.00% | 0.00% | 0.00% | 0.03% | 0.00% | 0.00% | 0.00% | 0.00% |
| SM4 - d18:1 / C16 | 0.00% | 0.04% | 0.02% | 0.00% | 0.16% | 0.02% | 0.07% | 0.31% | 0.12% | 0.02% | 0.00% | 0.03% | 0.00% |
| SM4 - d18:0 / C16 | 0.00% | 0.00% | 0.00% | 0.00% | 0.00% | 0.00% | 0.00% | 0.01% | 0.00% | 0.00% | 0.00% | 0.00% | 0.00% |

Table S2.
Human brain subjects utilized in this study for both control, AD and HS samples across eleven different brain regions.
